# Comprehensive analysis of Translationally Controlled Tumor Protein (TCTP) provides insights for lineage-specific evolution and functional divergence

**DOI:** 10.1371/journal.pone.0232029

**Published:** 2020-05-06

**Authors:** Namjin Koo, Ah-Young Shin, Sangho Oh, Hyeongmin Kim, Seongmin Hong, Seong-Jin Park, Young Mi Sim, Iksu Byeon, Kye Young Kim, Yong Pyo Lim, Suk-Yoon Kwon, Yong-Min Kim

**Affiliations:** 1 Korean Bioinformation Center, Korea Research Institute of Bioscience and Biotechnology, Daejeon, Republic of Korea; 2 Plant Systems Engineering Research Center, Korea Research Institute of Bioscience and Biotechnology, Daejeon, Republic of Korea; 3 Department of Biomedical Informatics, Center for Genome Science, National Institute of Health, KCDC, Choongchung-Buk-do, Republic of Korea; 4 Molecular Genetics and Genomics Laboratory, Department of Horticulture, College of Agriculture and Life Science, Chungnam National University, Daejeon, Korea; Laboratoire Oceanologique de Banyuls sur Mer, FRANCE

## Abstract

**Background:**

Translationally controlled tumor protein (TCTP) is a conserved, multifunctional protein involved in numerous cellular processes in eukaryotes. Although the functions of TCTP have been investigated sporadically in animals, invertebrates, and plants, few lineage-specific activities of this molecule, have been reported. An exception is in *Arabidopsis thaliana*, in which TCTP (AtTCTP1) functions in stomatal closuer by regulating microtubule stability. Further, although the development of next-generation sequencing technologies has facilitated the analysis of many eukaryotic genomes in public databases, inter-kingdom comparative analyses using available genome information are comparatively scarce.

**Methodology:**

To carry out inter-kingdom comparative analysis of TCTP, TCTP genes were identified from 377 species. Then phylogenetic analysis, prediction of protein structure, molecular docking simulation and molecular dynamics analysis were performed to investigate the evolution of *TCTP* genes and their binding proteins.

**Results:**

A total of 533 *TCTP* genes were identified from 377 eukaryotic species, including protozoa, fungi, invertebrates, vertebrates, and plants. Phylogenetic and secondary structure analyses reveal lineage-specific evolution of *TCTP*, and inter-kingdom comparisons highlight the lineage-specific emergence of, or changes in, secondary structure elements in TCTP proteins from different kingdoms. Furthermore, secondary structure comparisons between TCTP proteins within each kingdom, combined with measurements of the degree of sequence conservation, suggest that *TCTP* genes have evolved to conserve protein secondary structures in a lineage-specific manner. Additional tertiary structure analysis of TCTP-binding proteins and their interacting partners and docking simulations between these proteins further imply that *TCTP* gene variation may influence the tertiary structures of TCTP-binding proteins in a lineage-specific manner.

**Conclusions:**

Our analysis suggests that TCTP has undergone lineage-specific evolution and that structural changes in TCTP proteins may correlate with the tertiary structure of TCTP-binding proteins and their binding partners in a lineage-specific manner.

## Introduction

Translationally controlled tumor protein (TCTP) was first discovered in the late 1980s, [1, 2] and has subsequently been identified as a highly conserved protein expressed in all eukaryotic organisms [3–5]. Expression of the *TCTP* gene is tightly regulated in all tissues, at both the transcriptional and translational levels, particularly in mitotically active cells [6]. Additionally, TCTP protein levels vary in response to a wide range of extracellular signals and cellular conditions [7]. The TCTP protein is involved in many cellular processes, including cell growth [3, 4], protein synthesis, cell cycle progression [8], apoptosis [9–13], immune responses [14], and the regulation of pluripotency [15]. A number of proteins that interact with TCTP in various cellular pathways have also been reported, including TSC-22, BCL-xL, MCL1, RHEB, F-actin, tubulin, ELF3, Na-, and K-ATPase [16]. TCTP has been shown to play an important developmental role in both plants and animals. Homozygous knockout (*Tctp*^-/-^) mouse is embryonically lethal in mice, and a similar phenotype occurs in the plant, *Arabidopsis thaliana* [3, 17]. In *Drosophila*, organ-specific disruption of TCTP expression results in decreased cell numbers and defective cell growth [4]. TCTP has further been found to act as a regulator of tumor reversion and progression. In human prostate cancer cells, small interfering RNA (siRNA)-mediated silencing of *TCTP* leads to inhibition of cell growth and induction of apoptosis [18]. In addition, TCTP acts as an anti-apoptotic protein by interacting with MCL-1 and/or BCL-xL to block mitochondrial outer-membrane permeabilization occurring *via* the mitochondrial apoptosis-induced channel [10, 11].

The protein structure of TCTP from fission yeast (*Schizosaccharomyces pombe*) has been resolved by NMR spectroscopy, and that of the human protein was determined by X-ray crystallography [12, 19]. These analyses reveal three core domains comprised of nine β-strands, an α-helical hairpin, and a mobile loop, that are highly conserved between the human and yeast proteins. The H2-H3 helices of the α-helical hairpin also bear a structural resemblance to the H5-H6 helices of BCL-2 family proteins, such as BAX [12]. Amino acid sequence alignment of TCTP with BCL-2 family members indicates that the N-terminal region of TCTP contains a BH3-like domain that recognizes the BH3 groove of BCL-xL [20]. Calcium and microtubules also bind to the H2-H3 helices of the TCTP α-helical hairpin [8, 21]. The core domain of TCTP displays remarkable structural similarity to two families of proteins, namely the methionine sulfoxide reductases and mammalian suppressor of yeast SEC4 (MSS4), although it lacks major sequence similarity to other proteins. Further analyses of *TCTP* genes from various species, including lower-order taxa, have found that the number of *TCTP* genes per taxon is generally small, and all predicted structures harbor the G protein-binding pocket [22]. Critically, although the functions of specific regions of the TCTP protein have been elucidated, lineage-specific regions and their functions have not been investigated using comparative analysis.

Here, we report on an inter-kingdom analysis of TCTP proteins from 377 eukaryotes, including members of the protozoa, fungi, plant, and animal kingdoms. We found that *TCTP* gene copy numbers are higher in plants and mammals relative to other eukaryotes. Our structural prediction analysis demonstrated high conservation in the global tertiary structure of TCTP, although lineage-specific regions were also detected. Furthermore, a tertiary structure analysis of known TCTP-binding proteins, combined with molecular docking simulations, suggested that protein structures have changed in order to maintain TCTP binding. These findings are suggestive of a close evolutionary relationship between TCTPs and their respective binding proteins, indicating the functional diversification of TCTP proteins across eukaryotic taxa.

## Materials and methods

### Identification of *TCTP* genes

The ‘Gene Search’ section in Prometheus [23], a portal for inter-kingdom comparative analysis, was used to identify candidate *TCTP* genes using MSS4/translationally controlled tumor-associated TCTP (IPR011323) and family entry of translationally controlled tumor protein (IPR018105) as queries. A total of 533 candidate *TCTP* genes were identified, which included 117 sequences from 119 species of fungi, 54 sequences from 48 species of invertebrates, 109 sequences from 53 species of plants, 54 sequences from 46 species of protozoa, 157 sequences from 74 species of mammals, and 40 sequences from 39 species of other vertebrates. To eliminate partial and redundant sequences, candidate proteins were filtered by sequence length assigned to the TCTP domain and alternative splicing in their respective genes. The curated sequences were trimmed by matching the range for the TCTP domain to minimize the effects of regions outside the TCTP domain in further analysis. Then, we carried out blastp analysis using the NCBI NR database as subject and non-matched proteins were removed (S1 File).

For classification of eukaryotes, we used arbitrary organismal divisions, such as fungi, invertebrates, plants, protozoa, vertebrate mammalians, and vertebrate others. We note that organismal divisions do not adequately reflect NCBI taxonomy. Rather, these merely serve as a convenient way of dividing our dataset into smaller categories that include low-level taxonomic rank, such as a subphylum.

### Construction of phylogenetic tree

Evolutionary history was inferred using the minimum evolution (ME) method [24]. The sum of branch lengths in the optimal tree was 53.54. The phylogenetic tree was drawn to scale, with branch lengths in the same units as those of the evolutionary distances used to infer the tree. Evolutionary distances were computed using the number of differences method in units of number of amino acid differences per sequence. The ME tree was then searched using the Close-Neighbor-Interchange algorithm. The Neighbor-Joining algorithm [25] was used to generate the initial tree. This analysis involved 490 amino acid sequences, and all positions with less than 95% site coverage were eliminated. That is, alignment gaps, missing data, and ambiguous bases exceeding 5% were not allowed at any position. Evolutionary analyses were conducted in MEGA X [26].

### Multiple sequence alignment and protein sequence conservation

To calculate the best match for the given sequence in organismal divisions, we performed multiple sequence alignment (MSA) using ClustalW [27] for protein sequences classified by organismal division. The MSA was measured using the following parameters: scoring matrix = BLOSUM 62, opening gap penalty = 10, end gap penalty = 10, extending gap penalty = 0.05, and separation gap penalty = 0.05. Protein sequence alignment was used to measure conservation of amino acids in the web-based application WebLogo [28], the sequence logos from which indicate sequence conservation ratio, representing the relative frequency of each amino acid at individual positions within organismal divisions.

### Identification of TCTP-binding proteins

For the identification of TCTP-binding proteins, 32 known TCTP-binding partners were identified from literature surveys [16], and 22 proteins were found using either the STRING 10 database [29] with a minimum confidence score of interaction of 400, or supplied by UniProt [30]. We then curated a list of 40 binding proteins by combining the lists from literature and the database (Table 1). The sequences of these binding proteins were then retrieved from the reviewed Swiss-Prot database [30].

**Table 1:** Identification and ratio of TCTP-binding proteins (%).

Function	Genes of Binding Proteins	Fungi	Invertebrates	Plants	Protozoa	Mammals	Vertebrate others
Cytoskeleton/Mitotic Machinery	F-actin	100.0 (118/118)	100.0 (48/48)	98.0 (52/53)	100.0 (46/46)	100.0 (75/75)	97.0 (38/39)
	α-Tubulin	100.0 (118/118)	100.0 (48/48)	98.0 (52/53)	100.0 (46/46)	100.0 (75/75)	97.0 (38/39)
	CHFR	0.0 (0/118)	21.0 (10/48)	0.0 (0/53)	28.0 (13/46)	77.0 (58/75)	41.0 (16/39)
	β-Integrin	3.0 (3/118)	100.0 (48/48)	0.0 (0/53)	0.0 (0/46)	100.0 (75/75)	97.0 (38/39)
DNA Processing/DNA Repair	SC35	100.0 (118/118)	100.0 (48/48)	98.0 (52/53)	100.0 (46/46)	100.0 (75/75)	97.0 (38/39)
	ATM	1.0 (1/118)	33.0 (16/48)	9.0 (5/53)	0.0 (0/46)	100.0 (75/75)	92.0 (36/39)
RNA, Ribosome, Protein Biogenesis	EIF3	0.0 (0/118)	92.0 (44/48)	0.0 (0/53)	7.0 (3/46)	100.0 (75/75)	97.0 (38/39)
	EF1-α	99.0 (117/118)	95.0 (46/48)	98.0 (52/53)	93.0 (43/46)	99.0 (74/75)	97.0 (38/39)
	EF1-β	97.0 (115/118)	85.0 (46/48)	0.0 (0/53)	7.0 (3/46)	100.0 (75/75)	94.0 (37/39)
	SNX6	100.0 (118/118)	97.0 (47/48)	96.0 (51/53)	41.0 (19/46)	100.0 (75/75)	97.0 (38/39)
GTPases	TSC-22	0.0 (0/118)	92.0 (44/48)	89.0 (47/53)	0.0 (0/46)	100.0 (75/75)	97.0 (38/39)
	BCL-xL	3.0 (3/118)	13.0 (6/48)	0.0 (0/53)	0.0 (0/46)	99.0 (74/75)	95.0 (37/39)
	BAX	0.0 (0/118)	100.0 (48/48)	0.0 (0/53)	0.0 (0/46)	100.0 (75/75)	97.0 (38/39)
p53 Axis	MDM2	0.0 (0/118)	6.0 (3/48)	0.0 (0/53)	0.0 (0/46)	95.0 (71/75)	92.0 (36/39)
	p53	0.0 (0/118)	90.0 (43/48)	0.0 (0/53)	0.0 (0/46)	100.0 (75/75)	97.0 (38/39)
	RHEB	78.0 (92/118)	100.0 (48/48)	43.0 (23/53)	28.0 (13/46)	100.0 (75/75)	97.0 (38/39)

*Numbers of species corresponding to the binding protein are shown in parentheses.

### Prediction of tertiary structures and model refinement

To predict the tertiary structures of the TCTP, elongation factor 1-alpha 1 A (EF1A1), and Ras-related nuclear (RAN) proteins, we performed five steps of homology modeling (S1 Fig), including refinement of predicted models. To ensure high accuracy, it is important to identify suitable templates for prediction. Thus, in the first step, the target protein was used as a query to search for templates, in BLASTP [31] of the Protein Data Bank (PDB) [32], with an e-value cutoff ≤0.001. Templates with ≥35% sequence identity to each target sequence were chosen (S1 File).

In the second step, MSAs between potential templates and the target proteins were performed using the T-COFFEE (Expresso) program [33]. In cases where a template lacking structural information presented in a target protein, we used multiple templates for alignment to reinforce partial conformational regions.

In the third step, homology modeling was performed using MODELLER 9v17 [34] in order to construct a tertiary structure model for a target protein in each species. The predicted models were optimized by two distinct processes, spatial restraint and energy minimization, performed by the MODELLER program. To improve protein structure predictions, we performed 100 iterations of model assessment. Next, in the fourth step, a total of 100 predicted models were generated for the TCTP protein of each species, and a quality check of each protein structure was performed to ensure selection of a high-quality model. The best model from these ensembles was selected using three metrics: Ramachandran score, clash score, and normalized Discrete Optimized Protein Energy (nDOPE) score. The best model candidates possessed Ramachandran scores >90%, clash scores ([clashlist atoms]/[all atoms]×100) <5%, as determined by the MolProbity program [35], and nDOPE scores <0, as calculated using the MODELLER package [34]. The final best model for each protein was chosen according to the lowest nDOPE score.

In the final step, refinement of the best model was performed using the ModRefiner algorithm in the I-tasser package [36] over 20 iterations. This process reduces the energy score, modifies atomic clash, and adjusts for outliers in the Ramachandran plot. In total, 403 TCTP proteins from 327 species, 906 EF1A1 proteins from 251 species, and 344 RAN proteins from 195 species were used for further analyses (S2 File).

### Prediction of secondary structure

From the validated tertiary structures, assignment of secondary structure was performed using Database of Secondary Structure assignments for all Protein (DSSP) [37]. Assigned secondary structures were converted into continuous strings of secondary architectures with coils not shown. For the comparison of secondary structures and consensus sequences, the predicted secondary structures were aligned with the result of the MSA from ClustalW [27]. We have shown a representative pattern of secondary structures from each organismal division, in which the numbers of the secondary structures are shown at a maximum.

### Calculation of chemical properties for individual amino acids in TCTP proteins

To compare differences in TCTP secondary structures among organismal divisions, we investigated the properties for polarity (PO) and electronic charge (EC) of amino acids. They were calculated by prediction of properties for PO and EC of side chains at individual amino acids. The PO and EC values of TCTP proteins were assigned into score tables EC for the 20 amino acids [38], and we then calculated the average scores of PO and EC at individual residues in the MSA within organismal divisions. These properties of individual residues were shown as a heatmap, which was combined with the secondary structures and sequence conservation patterns.

### Comparison of tertiary structures of TCTP-binding proteins and their counterparts

To compare the tertiary structures of TCTP, EF1A1, and RAN proteins among organismal divisions, all predicted protein structures were superimposed, and the distance of each residue from the corresponding residue of the reference protein (TCTP: NP_003286.1 *Homo sapiens*, EF1A1: NP_001949.1 *H*. *sapiens*, RAN: NP_006316.1 *H*. *sapiens*) was measured by SHEBA 3.0 [39]. The amino acid distances were transformed into average root-mean-square deviation (RMSD) values in each organismal division, and represented in a plot (S2 Fig). In this plot, a high RMSD distance value indicates regions that are variable compared to the reference protein. The proteins that displayed the highest RMSD values in each organismal division were selected as representative tertiary structures for further analysis.

### Prediction of protein-protein interactions by molecular docking simulation

To predict protein-protein interactions, we performed a two-step docking simulation, which considers protein flexibility. The first step entailed a rigid-body docking simulation to investigate binding sites for protein-protein complex formation using the PatchDock package [40] with the following parameters: complex type = normal, and sampling method = high accuracy sampling. In this step, both rigid-docking and transformation results were obtained, and transformation results were used for subsequent analysis. The second step involved a flexible docking simulation using the FiberDock v.1.0 program [41] to investigate and refine flexible regions in protein-protein interactions. To enhance the accuracy of flexible-docking simulations, Normal Mod Analysis (NMA) was performed with 500 iterations using the following parameters: receptor, bound, side-chain optimization, and full side-chain optimization. To obtain the best model after conformational changes, we selected a model that displayed high scores in shape complementarity and energy function.

### Molecular dynamics simulation

To evaluate the stability of representative predicted protein structures and heterocomplexes containing TCTP and its binding partners identified from the molecular docking simulation, molecular dynamics (MD) simulations were performed using Amber 16 and AmberTools17 [42]. First, the complex system energy was minimized to relieve steric interactions that could interfere with dynamics: force field = ff14sb, and maximum cycle = 30,000 cycles. Next, the Langevin thermostat was used to maintain the temperature at 298K for 200 ns using the following parameters: nstlim = 100,000,000, dt = 0.002, and nstlim*dt = 200,000 ps. Calculation of RMSD, root-mean-square fluctuation (RMSF) and potential energy were carried out using CPPTRAJ analysis, and the results were visualized in Chimera [43].

## Results

### Inter-kingdom TCTP variants indicate a specific expansion in mammals and plants

**Fig 1:**
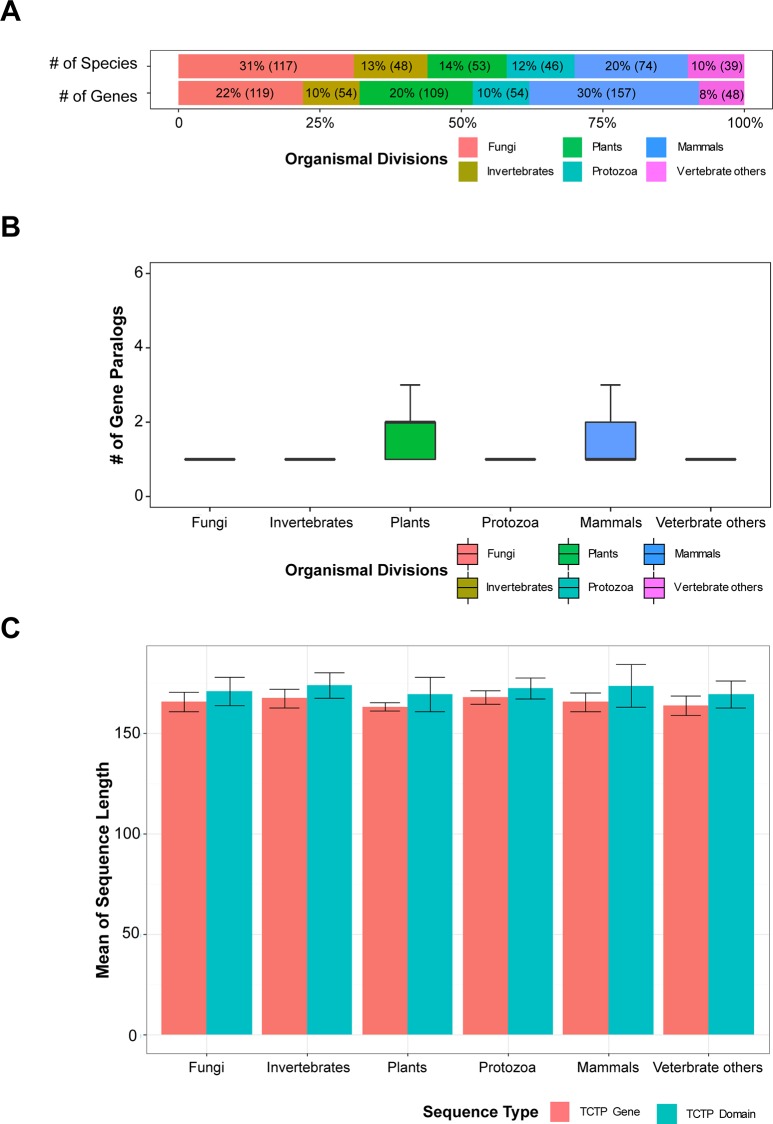
Identification of *TCTP* genes. (A) Distribution of *TCTP* genes across organismal divisions. (B) *TCTP* copy numbers in each species; *x*- and *y*-axes indicate organismal division and copy numbers, respectively. (C) Comparison of mean length of *TCTP* gene and TCTP domain; *x*- and *y*-axes indicate organismal division and sequence length (aa), respectively.

To carry out an inter-kingdom analysis of *TCTP* genes, we identified a wider set of candidate TCTP genes using Prometheus [23] with two IPR terms for MSS4/translationally controlled tumor-associated TCTP (IPR011323) and family entry of TCTP (IPR018105) in the three domains of life: Archaea, Bacteria, and Eukarya. *TCTP* genes were only detected in Eukarya; these 533 candidate genes were used for further analyses (Fig 1A). A previous study indicated that *TCTP* copy number per taxon is generally small [22]; however, here, we found that *TCTP* copy number differed among organismal divisions (Fig 1B). Specifically, our results revealed that most fungal, invertebrate, and protozoan genomes contain a single copy of the *TCTP* gene, whereas vertebrate and plant genomes harbor multiple copies. Interestingly, *TCTP* paralogs were detected in mammalian genomes (hereafter, “mammals”) but were not detected in other vertebrate genomes (hereafter, “vertebrate others”) (Fig 1B). These data suggest that *TCTP* paralogs emerged from duplication events that occurred in mammalian genomes after divergence from a common ancestor.

In previous studies, the length of TCTP was found to be approximately 170 residues and coverage of TCTP domains (IPR018105, IPR034737) was over 90% [5, 22]. However, here, we identified TCTP proteins greater than 200 residues in length. We note, however, that the accuracy of identification can be affected by genome annotation quality, and thus, these *TCTP* genes encoding proteins > 200 residues in length may result from mis-annotated genes. To account for this and remove mis-annotated and partial genes, we investigated domain architecture and coverage of TCTP domains in the *TCTP* genes we identified (Fig 1C). This curation showed that the average protein length is approximately 170 residues, and coverage of the TCTP domain in identified *TCTP* genes is approximately 90%. Collectively, these data suggest that *TCTP* genes emerged before the divergence of Eukarya and were sustained in small copy numbers during the evolution of each eukaryotic clade.

### Phylogenetic and domain conservation with secondary structure analyses indicate lineage-specific evolution of *TCTP*

**Fig 2:**
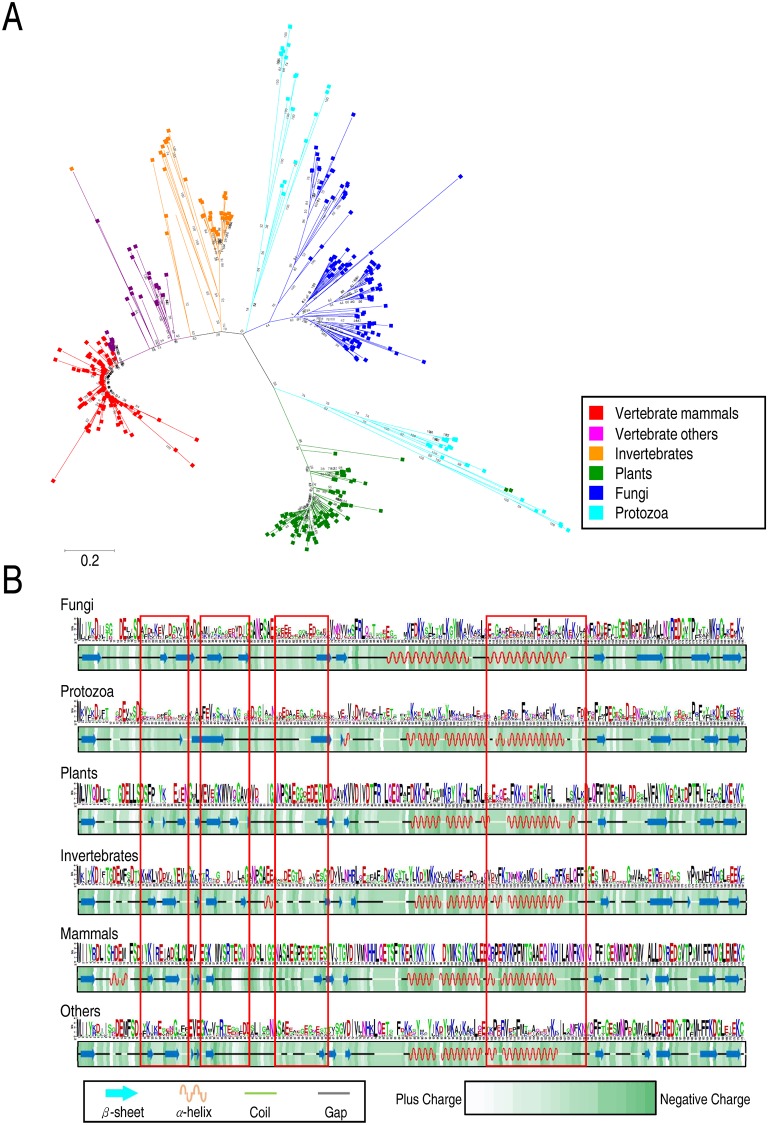
Phylogenetic analysis and domain conservation of *TCTP* genes in each organismal division. (A) The evolutionary history of *TCTP* genes was inferred using the minimum evolution method. (B) Conservation ratios for each amino acid are shown as a heat map, and a code indicating the degrees of conservation is shown in the right panel. Representative secondary structures for TCTPs from each organismal division are shown in the heat map. Lineage-specific domain deletion or emergence is indicated by colored boxes.

To investigate the evolution of *TCTP* genes, a phylogenetic analysis was performed using MEGA X (Fig 2A) [26]. We found that the 533 *TCTP* genes from six independent, taxon-specific clades, suggesting lineage-specific evolution of *TCTP* genes. *TCTP* genes in mammals and other vertebrates cluster into four independent clades. These patterns may reflect the recent duplication of genes in mammals, which presumably led to multiple copies of *TCTP* gene in these organisms. Sequence conservation was assessed in terms of the average conservation rate over all positions based on sequences using WebLogo [28], and secondary structures of TCTP variants were investigated based on predicted structures (Fig 2B). These data show high sequence similarities of *TCTP* genes across kingdoms, although lineage-specific secondary structures and conserved sequences were also detected. Interestingly, some *TCTP* genes in red algae and invertebrates *Hydra vulgaris* were clustered in the protozoan clade, whereas green algae *TCTP* genes were found in plant clades. These data suggest the possible horizontal gene transfer of *TCTP* between red algae or invertebrates and protozoa.

In previous studies reporting the amino acid sequence alignment of representative TCTP variants, nine residues (M1, E12, D15, S50, L74, K89, E134, P154, and K167) were found to be absolutely conserved, and six additional amino acids were mismatched in only one sequence, indicating a conservation of nearly 9% of amino acids [7, 22]. In addition, the lineage-specific function of TCTP in *A*. *thaliana*, wherein it promotes stomatal closure in response to water deficit [5], and a lineage-specific presence/absence of the binding protein, BCL-xL, were reported. These results are suggestive of lineage-specific evolution and functional roles of *TCTP*. To investigate this possibility, analysis of PO, EC, protein sequence conservation, and secondary structure analysis of TCTP proteins were performed (Fig 2, S3 and S4 Figs). To carry out these analyses, we investigated sequence conservation and chemical properties such as PO and EC based on the sequences of TCTP variants. These were generated from sequence-based multiple alignment by organismal division (Fig 2B, S3 and S4 Figs). The results revealed relatively high sequence conservation in plants, mammals, and other vertebrates compared to fungi, protozoa, and invertebrates. Conservation score was assessed by the average conservation rate over all positions in TCTP genes, and protozoa possessed particularly low sequence conservation (S5 Fig). Furthermore, lineage-specific sequence variations were detected in long coils, double α-helices, and β-sheets in the N-terminal regions of TCTP (Fig 2B, highlighted boxes). Next, we investigated the secondary structures of TCTP among organismal divisions. There are two methods for the prediction of secondary structures of proteins: sequence- and structure-based prediction. We performed prediction of secondary structures using these two methods and compared secondary structures predicted by these two methods. The results revealed approximately 80% structural similarity, with regions showing structural variation corresponding to those showing sequence variations. Further evaluation of these predictions using reference protein structures (*H*. *sapiens*: 1YZ1, *S*. *pombe*: 1H6Q, and *Caenorhabditis elegans*: 2LOY) suggested that structure-based predictions were more precise than sequence-based predictions (S3 Fig). Investigation of chemical properties revealed that patterns of PO and EC were different among organismal divisions. PO and EC play important roles in the folding and function of proteins [44]. These results suggested lineage-specific roles for TCTP proteins, although their tertiary structures are similar. Collectively, the secondary structures of these proteins are conserved, but different patterns of PO and EC caused by sequence variations may have led to the lineage-specific evolution of TCTP proteins.

### Tertiary structure analyses indicate lineage-specific evolution and functional roles of TCTP

**Fig 3:**
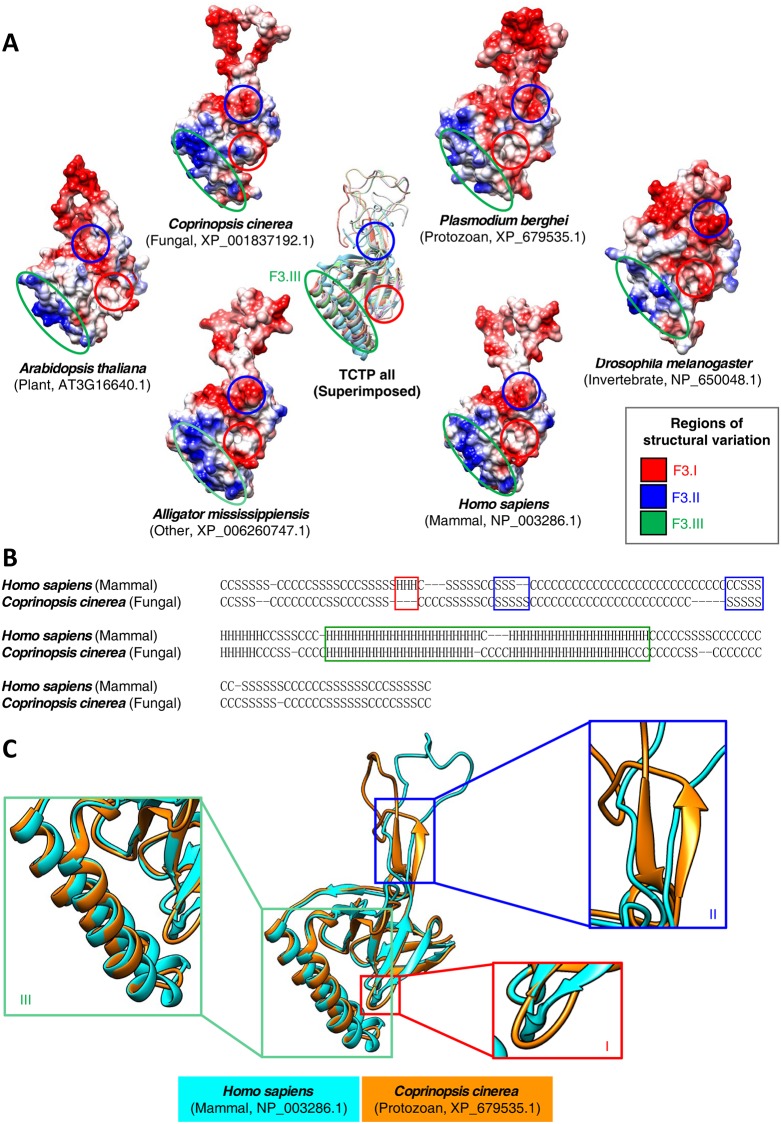
Prediction and alignment of TCTP tertiary structures. (A) Inter-organismal division comparison of TCTP tertiary structures, as illustrated by molecular electrostatic potential surface (MEPS) of TCTP structures are shown. Tertiary structures were predicted using BLAST, T-COFFEE (Expresso), and MODELLER 9v17 and visualized using Chimera. (B) Secondary structure alignment of human (NP_003286.1) and fungal TCTPs (*Coprinopsis cinerea*, XP_001837192.1) using Secondary Structure Element Alignment (SSEA) [60]. (C) Comparison of the predicted tertiary structures from the human and fungal TCTP proteins shown in (B). Three different regions are shown as enlarged boxes.

Since the elucidation of the TCTP structure from *S*. *pombe* (fungi; PDB 1H6Q and 1H7Y), 9 wild-type or mutant TCTP structures have been investigated in species such as *H*. *sapiens* (mammals; PDB 1YZ1, 2HR9, 3EBM and 4Z9V), *C*. *elegans* (invertebrates; PDB 2KWB and 2LOY), *Plasmodium falciparum* (protozoa; PDB 3P3K), *Plasmodium knowlesi* (protozoa; PDB 1TXJ), and *Nanochloropsis oceantia* (plants, PDB 6J2Y). Here, we performed homology modeling, including model refinement, and compared the predicted tertiary structures for all identified TCTPs (Fig 3, S1 Table). To enhance the accuracy of homology modeling, we used multiple templates for each prediction. These homology modeling data indicated that template combinations were similarly composed in all organismal divisions (S2 Table).

Refined models showed similar or higher quality compared to known structures present in PDB, whereas non-refined models showed lower quality than known structures (S3 Table). The structural clustering of proteins further indicated distinct groups corresponding to organismal division (S4 Table), and these data suggest that our pipeline generates high-quality models to detect structural differences among organismal divisions.

Based on the predicted tertiary structures, we found that TCTPs belonging to the same organismal division formed two to four independent clusters. Furthermore, global tertiary structures among TCTPs are similar (S5 and S6 Tables), although variations in two core domains, nine β-sheets, and α-helical hairpin structures were detected in each organismal division (Fig 3A). These results consist with previous study about structural comparison using *N*. *oceantia* TCTP (NoTCTP) and other TCTP proteins [45]. These variations were consistent with sequence variations (Fig 2B) and solvent accessibility was predicted based on the tertiary structures of TCTPs (Fig 3A). Furthermore, hydrophobicity analysis revealed distinct hydrophobic surfaces in three different regions.

For further comparison among organismal divisions, secondary structure alignment was performed using human and fungal (*Coprinopsis cinerea*) TCTPs (Fig 3B). Our results revealed minor structural variations in three core domains, and sequence variations correlate with the tertiary structures of both TCTPs (Fig 3C). A single-turn α-helix can be detected in almost all vertebrate TCTPs (Figs 2B and 3C, Box I), and the lengths of helices and β-sheets are different in human and fungal TCTPs (Fig 3, Box II and III). In addition, RMSD distances between individual amino acids in each TCTP were calculated using the human TCTP (*H*. *sapiens*: NP_003286.1) as a reference (S2 Fig). In this plot, a high RMSD distance indicates the presence of regions that are variable compared to the reference protein. Thus, our data imply that tertiary structure variations can also be detected in TCTPs from different organismal divisions. These structural variations may correlate with the affinity of TCTPs for their respective binding proteins or for cations, such as sodium or calcium.

### Molecular docking simulation between TCTP and its binding proteins

To investigate the possibility that the structural variations of TCTPs correlate with the tertiary structures of their respective binding proteins, we first identified 40 TCTP-binding proteins using the STRING 10 database [29] and a literature survey [16]. Among these, 16 were selected as representative genes, and inter-kingdom identification was performed based on their domain architectures using Prometheus (Table 1) [23]. A total of 16 representative TCTP-binding proteins were assigned to five pathways, as described in a previous study [16]. We found that genes such as F-actin, α-tubulin, and *EF1A1* were present in all genomes analyzed, whereas lineage-specific genes, such as *BCL-xL*, *ELF3*, and *TSC-22*, were detected only in individual organismal divisions. TCTP-EF1A1 interaction were reported by previous studies as a TCTP-EF1A1-EF1A2 complex [46–49]. These data imply a correlation between lineage-specific regions of TCTP and the presence/absence of TCTP-binding proteins.

Consistent with previous research, one well-known TCTP-binding protein, BCL-xL, and its binding protein BAX, was not detected in plants [50]. It was previously found that a BH-3-like domain (11 to 31 residues) in the N-terminal region of TCTP is involved in binding to BCL-xL, activates the anti-apoptotic function of BCL-xL. Interaction between BCL-xL and the BH3-like domain of TCTP is mediated by both hydrophobic and electrostatic interactions [20]. We therefore analyzed molecular electrostatic potential surface (MEPS) and hydrophobicity of a single-turn α-helix and its neighboring region in TCTPs (S6 Fig). TCTPs in each organismal division show different patterns of hydrophobicity and/or the distribution of charged amino acid residues. In vertebrate TCTPs, the single-turn α-helix and its neighboring region showed similar distributions of MEPS and hydrophobicity. However, TCTPs in other organismal divisions displayed different MEPS and hydrophobicity distributions. Thus, these data indicate that the single-turn α-helix in vertebrate TCTPs might have the potential to refold into a three-turn α-helix. Furthermore, conservation of structures in the single-turn α-helix in vertebrates may be affected by the presence of BCL-xL.

**Fig 4:**
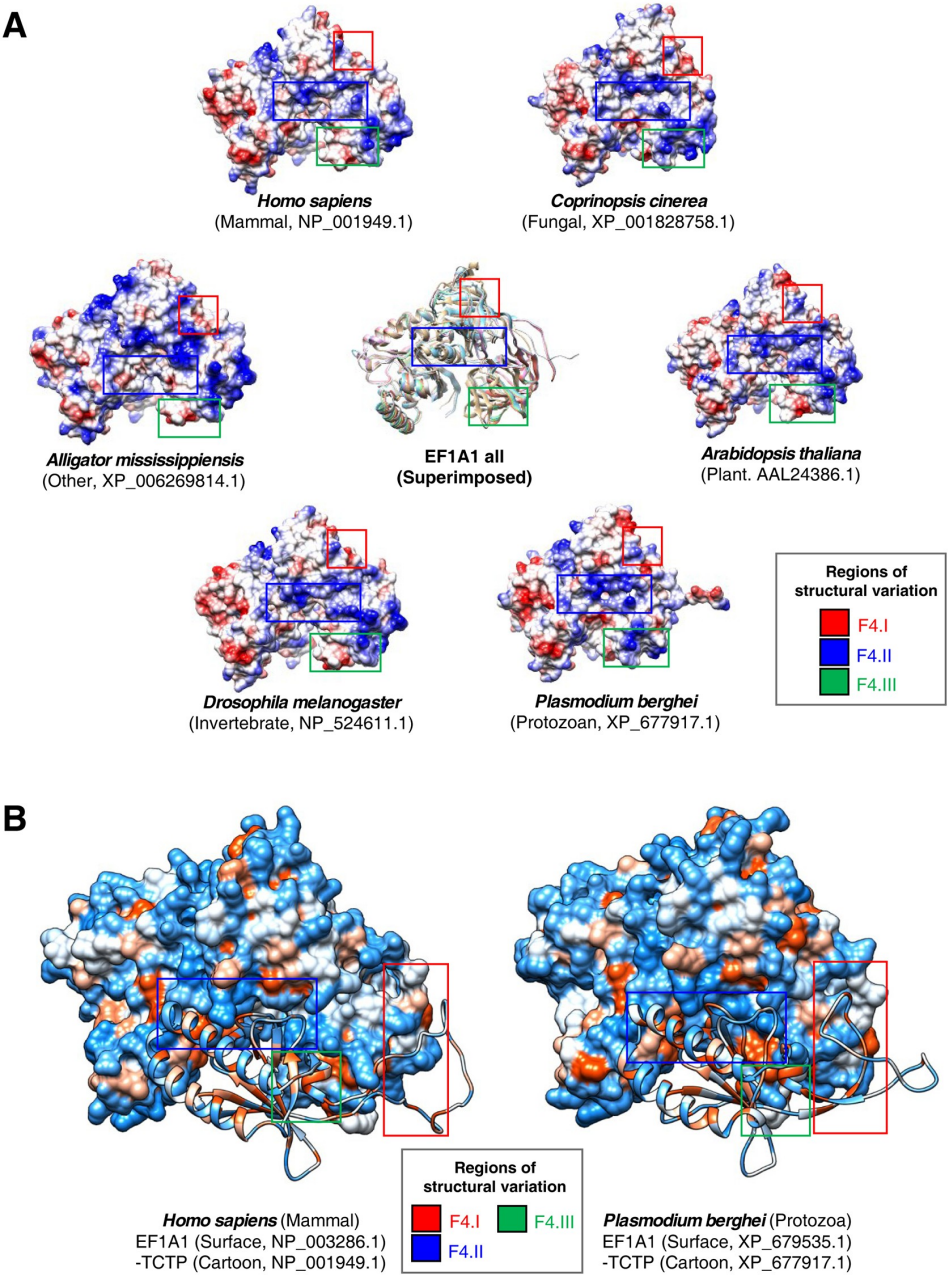
Predicted tertiary structures for human and protozoan EF1A1 and docking simulations with TCTP. (A) Tertiary structures of the TCTP-binding protein, EF1A1, from six organismal divisions were predicted by the homology modeling method and visualized using Chimera as molecular electrostatic potential surface (MEPS) models. Superimposed models are shown in the center. (B) Docking simulations between TCTP and EF1A1 were performed using the PatchDock and FiberDock1.0. Representative results, docking between human and protozoa TCTP and EF1A1, are shown here with molecular surface hydrophobicity of EF1A1 and TCTP is shown as a cartoon.

The tertiary structures of binding proteins may also be correlated with TCTP variations. To investigate this possibility, we predicted the tertiary structure of EF1A1, which binds TCTP and promotes the GTP-dependent binding of aminoacyl-tRNA to the A-site of ribosomes during protein biosynthesis, using BLAST [51] and MODELLER 9v17 (Fig 4, S7 Table) [52]. Our results reveal numerous variations across six organismal divisions in EF1A1 structure in the C-terminal flexible β-sheet region involved in TCTP binding (Fig 4A, Red box). In TCTPs, the EF1A1-binding site varied across all organismal divisions including plants and protozoa (Fig 2B). Thus, these data suggest that variation in the EF1A1-binding site of TCTP may correlate with the structure of the C-terminal flexible β-sheet region in EF1A1. Alternatively, these data also suggest the possibility that the variation in EF1A1 promotes variation in the corresponding binding site of TCTP. Molecular docking simulation between TCTP and EF1A1 further revealed distinct differences in the heterocomplex-binding site between humans and protozoa (Fig 4B). The protein structure of EF1A1 is similar to that of EF1A2 protein; although surface electrostatic properties differ [53], the structure of domain I and II are comparable. However, the electrostatic difference may affect binding affinities of EF1A1 and EF1A2 to TCTP. Previous docking simulation results are consisted with our findings [53], as is FLAG immunoprecipitation indicating that EF1A2 shows higher affinity for TCTP than EF1A1 [49]. Specifically, the human heterocomplex is compact relative to that of protozoa, which contains a flexible β-sheet region from EF1A1 and a flexible long-coil region from TCTP (Fig 4B), potentially providing structural flexibility to the protozoan heterocomplex.

**Fig 5:**
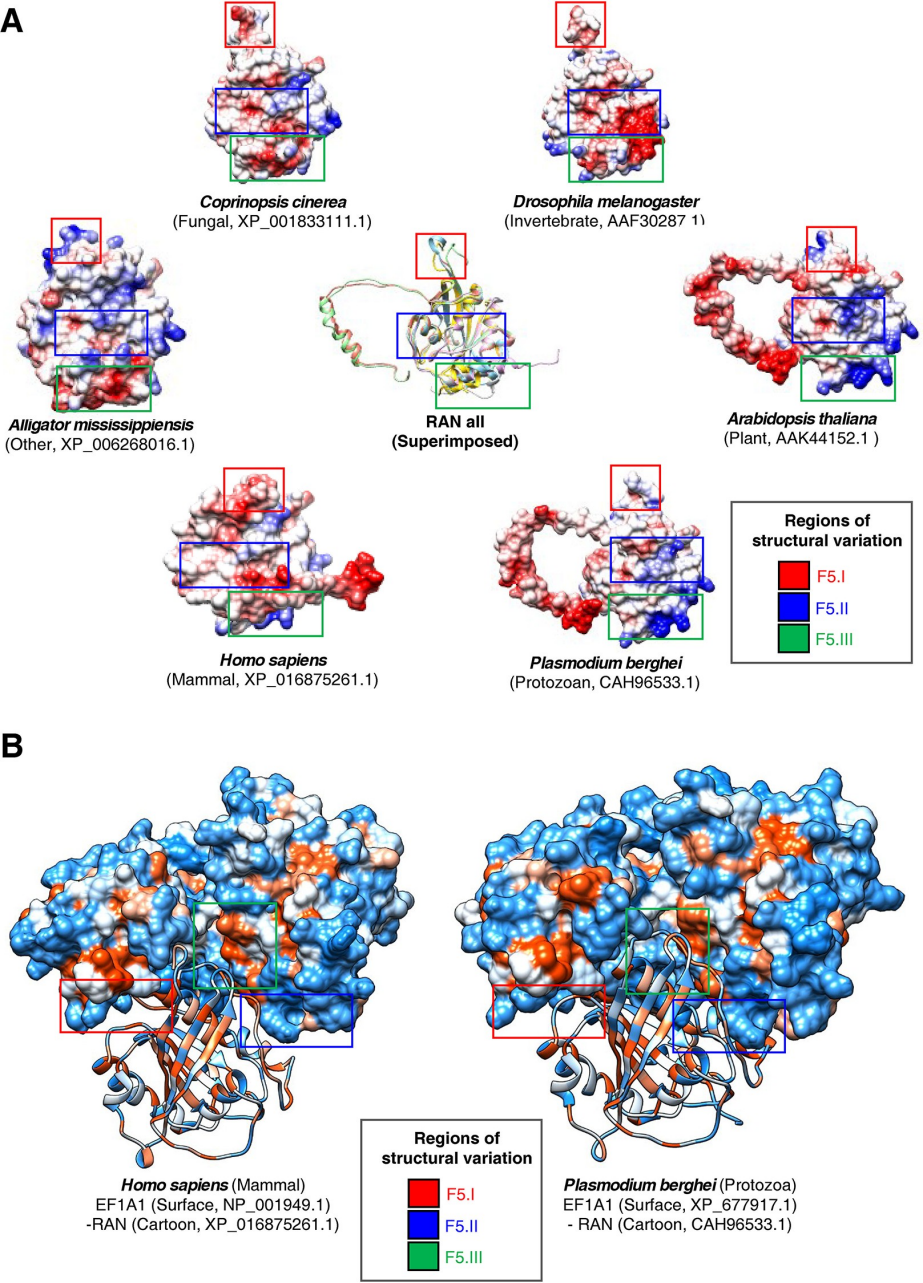
Tertiary structures of human and protozoa RAN proteins and their predicted interaction with EF1A1. Tertiary structures of the EF1A1-binding protein, RAN, from different organismal divisions were predicted by the homology modeling method with multiple templates and visualized using Chimera. (A) Comparison of RAN proteins from six organismal divisions shown molecular electrostatic potential surface (MEPS). Superimposed models are shown in the center. (B) Docking simulations between EF1A1 and RAN were performed using PatchDock and FiberDock1.0. Predicted EF1A1-RAN heterocomplexes in *H*. *sapiens* and *P*. *berghei* are shown with molecular surface hydrophobicity of RAN and EF1A1 displayed as a cartoon.

We next predicted the tertiary structures of RAN, a known EF1A1-binding protein, from six organismal divisions (Fig 5, S8 Table). From this analysis, we detected two flexible regions and one instance of length variation in the β-sheet among organismal divisions (Fig 5A). Further molecular docking simulation revealed that the C-terminal flexible region of RAN binds to the flexible β-sheet region of EF1A1 (Fig 5B). A comparison of the EF1A1-RAN complex between mammals and protozoa indicated that the human complex has a different structural interface between the binding site and solvent properties than the protozoan complex (Fig 5B). Specifically, the solvent accessibility of amino acids in the EF1A1 and RAN structures represent differences in the distribution for binding regions of EF1A1-RAN (Fig 5B, Box I—III). These differences may result in shorter distances between residues in the interaction region in the EF1A1-RAN complex of *Plasmodium berghei* than in that of *H*. *sapiens*. Changes in solvent accessibility lead to different interactions of the amino acids at the EF1A1-RAN complex binding site among organismal divisions (Fig 5A).

### TCTP structural variations may correlate with structural changes of TCTP-binding proteins

**Fig 6:**
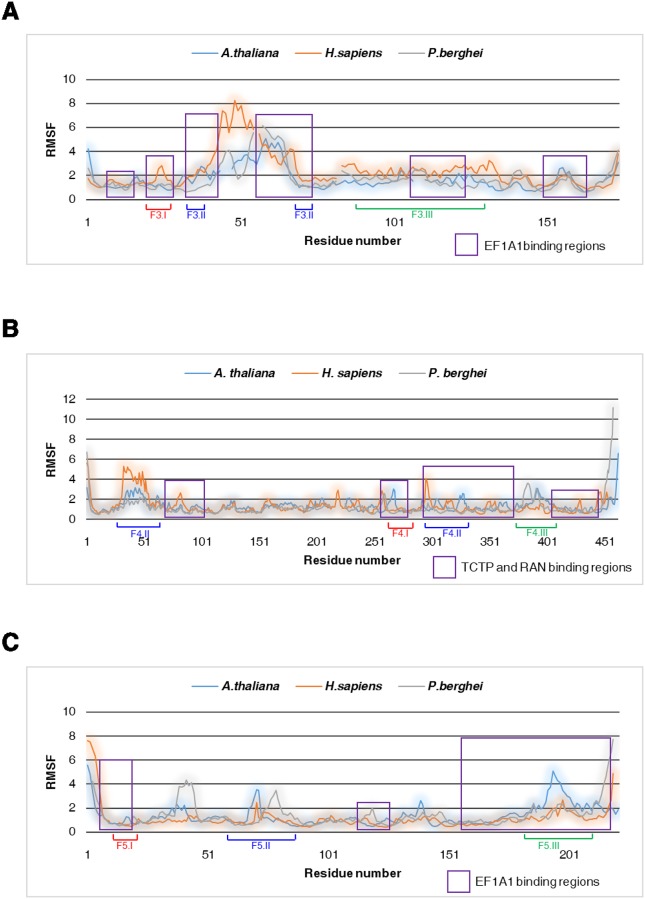
Root mean square fluctuation (RMSF) from molecular dynamics (MD) for flexibility analysis. RMSF plots for (A) TCTP, (B) EF1A1, (C) and RAN proteins. The binding regions of each protein are shown as purple-colored boxes.

To evaluate the stability of homology models and complexes for the proteins, we performed MD simulations. First, we carried out MD simulations of individual TCTP, EF1A1, and RAN molecules from three representative species used in our molecular docking simulations (S7 and S8 Figs). Our results indicate that all predicted structures are maintained at equilibrium state with respect to RMSD value and total energy over 30,000 ps (30 ns). The RMSF data further show that mobile loops in TCTP are highly flexible among the three species. In TCTP, EF1A1-binding regions (Fig 6A, purple boxes; S9A Fig) are also flexible among different species (Fig 6A, S9A Fig). These patterns are also detected in the TCTP- and RAN-binding regions of EF1A1 (Fig 6B, S9B Fig), and in the EF1A1-binding regions of RAN (Fig 6C, S9C Fig). Further assessment of the distance matrix of intra-interaction in these proteins reveals structural differences in the TCTP, EF1A1, and RAN proteins among organismal divisions (S10 Fig). Thus, these differences in the flexibility of binding regions and intra-interaction in TCTP, EF1A1, and RAN proteins suggest a potential effect on the binding of these proteins to their respective partners.

Additional MD simulations of protein complexes were then carried out to evaluate the stability between TCTPs and their respective binding proteins. MD simulation output data were analyzed based on total energy (S11A Fig) and RMSD value (S11B Fig) for a time period of 200 nanoseconds (ns). We found that all TCTP-EF1A1 and EF1A1-RAN complexes are stable, with no drift in total energy over their respective RMSD value. The TCTP-EF1A1 complexes displayed a stable state at energy values of 3,200 to 3,700 kcal/mol and RMSD values of 3.5–3.8 Å, and the EF1A1-RAN complex reached convergence at energy values of 2,700 to 3,200 kcal/mol and RMSD values of 3.7–4.3 Å. From these results, we conclude that our protein structural prediction and molecular docking analyses are accurate and suitable for comparative analysis of protein structures and their functional roles. Collectively, our data suggest that the emergence of new lineage-specific genes such as *BCL-xL* or *BAX* in vertebrates may have correlate with structural variations in TCTP. Furthermore, these TCTP structural variations may correlate with structural changes in other binding proteins and their counterparts.

## Discussion

Since the first characterization of TCTP in tumor cells, many studies have characterized and elucidated the role of this protein. TCTP is multifunctional with roles in cell growth, protein synthesis, cell cycle progression, apoptosis, and immune responses. Most species in Eukarya harbor at least one *TCTP* gene, whereas the other two domains of life, Archaea and Bacteria, have no *TCTP* genes. In plants, various biological functions of *TCTP* genes have been reported. *AtTCTP1* plays important roles in drought tolerance [5] as well as embryo development [17]. *OsTCTP* plays a role in mercury tolerance in rice [54] and cassava *MeTCTP* gene involves in salt stress response [55]. Although almost all eukaryotes possess *TCTP* genes, copy numbers are generally low, but the whole genome duplication (WGD) events that occur in eukaryotic genomes, may affect copy numbers [56]. A previous study in plant genomes has suggested that WGD was followed by many gene deletions (homolog gene loss) that led to copy number variations [57, 58]. These results imply that *TCTP* gene copy numbers were strictly sustained in small numbers during evolution. Additionally, our results further reveal that *TCTP* copy numbers differ according to organismal division, with multiple copies present in plants and mammals (Fig 1B).

In plants, *Arabidopsis* contains two copies of *TCTP* genes, *AtTCTP1* (*At3g16640*), and *AtTCTP2* (*At3g05540*), the products of which share approximately 80% similarity at the amino acid level. AtTCTP1 is involved in regulating proliferation and development, as well as in the modulation of microtubule stability in response to water deficit [5]. Although *AtTCTP2* was thought to be a pseudogene [5, 17], recent studies have suggested functionality because its presence enhances *in vitro* plant regeneration [58]. Consistent with this evidence, a T-DNA insertion mutant of *AtTCTP2* (*SALK_045146*) shows a lethal phenotype during early development [58]. Furthermore, *AtTCTP1* mRNA from *AtTCTP2* mutants accumulates to higher levels than in wild-type plants, which implies functional redundancy between *AtTCTP1* and *AtTCTP2* [59]. However, the two genes show different expression patterns, and *AtTCTP1* could not rescue *AtTCTP2* knockout in plants [58]. These data suggest distinct roles for both genes with some degree of functional redundancy. Therefore, the multiple copies of *TCTP* present in plants and mammals may suggest subfunctionalization or neofunctionalization of duplicated *TCTP* genes in plants and mammals during evolution. Previous studies in *Arabidopsis* further reveal that AtTCTP2 has a deletion of 13 amino acids in the fifth β-sheet not present in AtTCTP1 (Fig 2B). This structural variation may be critical for the functional difference between the two *Arabidopsis* TCTP paralogs. Similarly, small sequence or structural variations in TCTPs may promote the emergence of different functional roles. Thus, the investigation of secondary and tertiary structures of identified TCTPs with sequence variations can elucidate functional differences in *TCTP* genes among organismal divisions. Further, investigations of sequence conservation pattern and conservation score also indicated that high conserved amino acids differ among organismal divisions (Fig 2B and S5 Fig), with relatively high sequence variation were detected in protozoa and fungi. These results suggest functional diversification of TCTP among organismal divisions.

In our study, a total of 11 TCTP templates were identified in the PDB, consisting of four mammalian proteins (PDB 1YZ1, 2HR9, 3EBM and 4Z9V), two fungal proteins (PDB 1H6Q and 1H7Y), two invertebrate proteins (PDB 2KWB and 2LOY), two protozoan proteins (PDB 1TXJ and 3P3K), and one plant protein (PDB 6J2Y). TCTP homology models were then predicted using these 11 templates (S2 Table). Then, predicted structures were calculated by considering the flexible integration of sequence identity and the frequency of secondary structural elements, including prediction of spatial restraints such as homology restraints, stereochemical restraints, and statistical restraints using MODELLER [34]. Finally, low-quality protein structures, as determined by Ramachandran and clash scores, were removed from the set of predicted protein structures.

Here, our secondary structure analysis revealed lineage-specific deletion and/or emergence of distinct secondary structures (Fig 2B), and further indicated differences in sequence conservation patterns among organismal divisions. Remarkably, a single-turn α-helix in the N-terminal region of TCTP resembles a BH3-like domain and plays an important role in binding to BCL-xL. In previous studies, a single-turn α-helix was detected in the BH3-like domain of TCTP [12], and this single-turn α-helix was refolded into a three-turn α-helix upon binding to BCL-xL [20]. In our current study, nearly all TCTPs in vertebrates contained a single-turn α-helix in their BH3-like domain, whereas only a small number of TCTPs from other organismal divisions displayed this feature (S9 Table). The hydrophobicity and MEPS of a single-turn α-helix and its neighboring region further revealed that vertebrate TCTPs display conserved hydrophobicity and MEPS compared to other organismal divisions (S6 Fig). Hydrophobicity and EC affect the folding and function of proteins [38]. Thus, these data suggest that almost all vertebrate TCTPs contain a single-turn α-helix with the potential to refold into a three-turn α-helix (S6 Fig).

Notably, an inter-kingdom investigation revealed that *BCL-xL* genes are only present in vertebrates (Table 1), suggesting that the presence of this binding partner can affect the conservation of structure and MEPS of a single-turn α-helix in vertebrate TCTPs. Furthermore, this conservation was associated with a vertebrate-specific role of TCTP as an anti-apoptotic protein. Additional secondary structure differences were also detected. Specifically, the β-sheet in front of the α-helix hairpin domain was altered in invertebrates and plants but unchanged in protozoa, fungi, and vertebrates implying functional differences and/or binding protein differences among organismal divisions. We further postulate that lineage-specific changes in this region of TCTP occurred independently in each organismal division, after divergence from a common ancestor; this hypothesis is supported by the region’s high sequence variation among organismal divisions (Fig 2B, S2 Fig).

Tertiary structure predictions for TCTP revealed a high level of conservation within organismal divisions, with only minor variations. However, TCTPs belonging to different organismal divisions showed relatively high differences in the structures of two core domains, specifically, the α-helix hairpin and nine β-sheets (Fig 2B). The distance matrix of intra-interaction also revealed structural differences in TCTPs (S10A Fig), and RMSF data further indicated that patterns of flexible regions in TCTPs caused by sequence variations are different among organismal divisions (Fig 6A). These structural variations might correlated with the corresponding tertiary structures of TCTP-binding proteins, leading to lineage-specific structural variations and/or functions of these binding proteins.

To investigate the influence of TCTP tertiary structure on its binding proteins, we analyzed the tertiary structure of EF1A1, which is known to interact with TCTP (Fig 4). Our results indicate that the tertiary structure of EF1A1 has substantial variations in the flexible β-sheet region (Fig 4A). Notably, the EF1A1-binding site in TCTP was found to be highly variable in plants and protozoa (Fig 2B); similarly, the corresponding TCTP-binding site in EF1A1 is also highly variable (Fig 4A). MD simulations reveal that in EF1A1 the TCTP- and RAN-binding sites are in flexible regions (Fig 6A and 6B). The matrix of intra-interaction indicates structural differences in EF1A1 among species (S7B Fig).

Our molecular docking simulations further demonstrated that heterocomplexes containing TCTP and EF1A1 differ in humans, plants, and protozoa (Fig 4B). These data suggest that the structural variations in TCTPs may correlate with the tertiary structures of its binding proteins, such as EF1A1. Accordingly, tertiary structure analyses with the EF1A1-binding protein, RAN, revealed that its EF1A1-binding regions also vary among organismal divisions (Figs 5A and 6C) In this case, molecular docking simulations indicated that the RAN-binding site for EF1A1 is in the flexible β-sheet region, identified as a TCTP-binding site (Fig 5B). These data suggest that structural changes in TCTPs may correlate with structural changes in TCTP-binding proteins, as well as additional proteins with which they interact (e.g. RAN). Furthermore, alterations in the tertiary structures of EF1A1, such as the flexible β-sheet region, can correlate with structural variation in TCTP-EF1A1 heterocomplexes and play lineage-specific roles among different organismal divisions. These data suggest that variations in TCTP may correlate with the tertiary structures of its binding partners and, consequently, the functional roles of TCTP-binding proteins.

## Conclusions

In the present study, we identified *TCTP* genes and TCTP-binding proteins from a wide range of species and performed a detailed analysis of their predicted protein structures. Our results suggest that *TCTP* genes evolved in a lineage-specific manner that included protein structural changes. Moreover, the tertiary structures of some TCTP-binding proteins, such as EF1A1 and its binding partner RAN, may be correlated with variations in TCTPs. Thus, we propose that structural changes in TCTPs may correlate with the tertiary structures and potentially the functionality of TCTP-binding proteins and their counterparts.

## Supporting information

S1 File (XLSX)

S2 File (XLSX)

S3 File (XLSX)

S1 Fig Pipeline for homology modeling and molecular docking of TCTPs and their binding protein. The pipeline divided into three parts and programs for each part were described in the rectangle with conditions.
(DOCX)

S2 Fig RMSD Distribution of amino acids in individual TCTPs from Human TCTP. The distance of each amino acids were measured using human TCTP as a control and average distance and standard deviation of the RMSD were calculated within each organismal divisions.
(DOCX)

S3 Fig Secondary structure prediction result using two methods. This figures viewing average secondary structure. To secondary structure predict, we using two method. First, we predicted sequence based secondary structure using psi-pred 3.4. Second, we predicted structure based secondary structure using DSSP.
(DOCX)

S4 Fig Combined result of multi analysis data. Polarity and secondary structure merging result.
(DOCX)

S5 Fig Conserved scores of TCTP genes. Conservation was assessed in terms of the average conservation rate over all positions in TCTP genes. The conservation score is the proportion of conserved amino acids in a window of size 10.
(DOCX)

S6 Fig Molecular Electrostatic Potential Surface (MEPS) and hydrophobicity of a single turn α-helix and its neighboring region. MEPS (left) and hydrophobicity molecular surface (right) of a single turn α-helix and its neighboring region in TCTPs from two representative species were visualized using Chimera.
(DOCX)

S7 Fig Time variation of the RMSD during the MD simulation period of 30,000 pico second (30 nS) for protein structure (TCTP, EF1A1, RAN) evaluation. Time evolutions of the backbone RMSD of TCTP (A), EF1A1 (B) and RAN(C) were shown. Colored-bars represent corresponding species.
(DOCX)

S8 Fig Time variation of total energy during the MD simulation period of 30,000 picosecond (30 nS) for protein structure (TCTP, EF1A1, RAN) evaluation. Time evolutions of (A) folding free energy of TCTP (A), EF1A1 (B) and RAN(C) were shown. Colored-bars represent corresponding species.
(DOCX)

S9 Fig Distribution of RMSF with pocket site for TCTP. EF1A1-binding region show a flexible property among *A*. *thaliana* (A), *H*. *sapiens* (B) and *P*. *berghei* (C). Purple-colored boxes indicate EF1A1 binding site.
(DOCX)

S10 Fig Distance matrix of intra-interaction among residues in a single molecule. The distance matrix of three representative species of TCTP (A), EF1A1 (B), and RAN (C) were shown and red circles represent difference of interacting residues.
(DOCX)

S11 Fig Estimated equilibration state and stability of MD simulation period of 200,000 pico second (200 nS) for complex structure (TCTP-EF1A1, EF1A1-RAN) evaluation. Time evolutions of (A) total energy of TCTP-EF1A1 and EF1A1-RAN, (B) the backbone RMSD of TCTP-EF1A1 and EF1A1-RAN. Each graph (A, B) include *A*. *thaliana*, *H*. *sapiens* and *P*. *berghei*.
(DOCX)

S1 Table Structure quality check of predicted protein structure about treat refinement by organismal divisions. (DOCX)

S2 Table Sub-chain number in the selected templates by each organismal division. (DOCX)

S3 Table Structure quality check between modeling structure and PDB. (DOCX)

S4 Table Structural clustering of TCTP proteins using MaxCluster program. (DOCX)

S5 Table Comparison of structural similarity (TMalign score) by organismal deviations. (DOCX)

S6 Table Structural similarity score of TCTP, EF1A1 and RAN. (DOCX)

S7 Table Structure quality check of representative EF1A1 protein structure by organismal divisions. (DOCX)

S8 Table Structure quality check of representative RAN protein structure by organismal divisions. (DOCX)

S9 Table Statistics of secondary structure among 3, 4 ß-sheet. (DOCX)
