# Supplementary material for: Comprehensive analysis of Translationally Controlled Tumor Protein (TCTP) provides insights for lineage-specific evolution and functional divergence
Source: PLoS One. 2020 May 6;15(5):e0232029. doi: 10.1371/journal.pone.0232029 (PMC7202613; doi:10.1371/journal.pone.0232029)
Supplement: S1 Fig — The pipeline divided into three parts and programs for each part were described in the rectangle with conditions. (DOCX) [file pone.0232029.s004.docx]

**
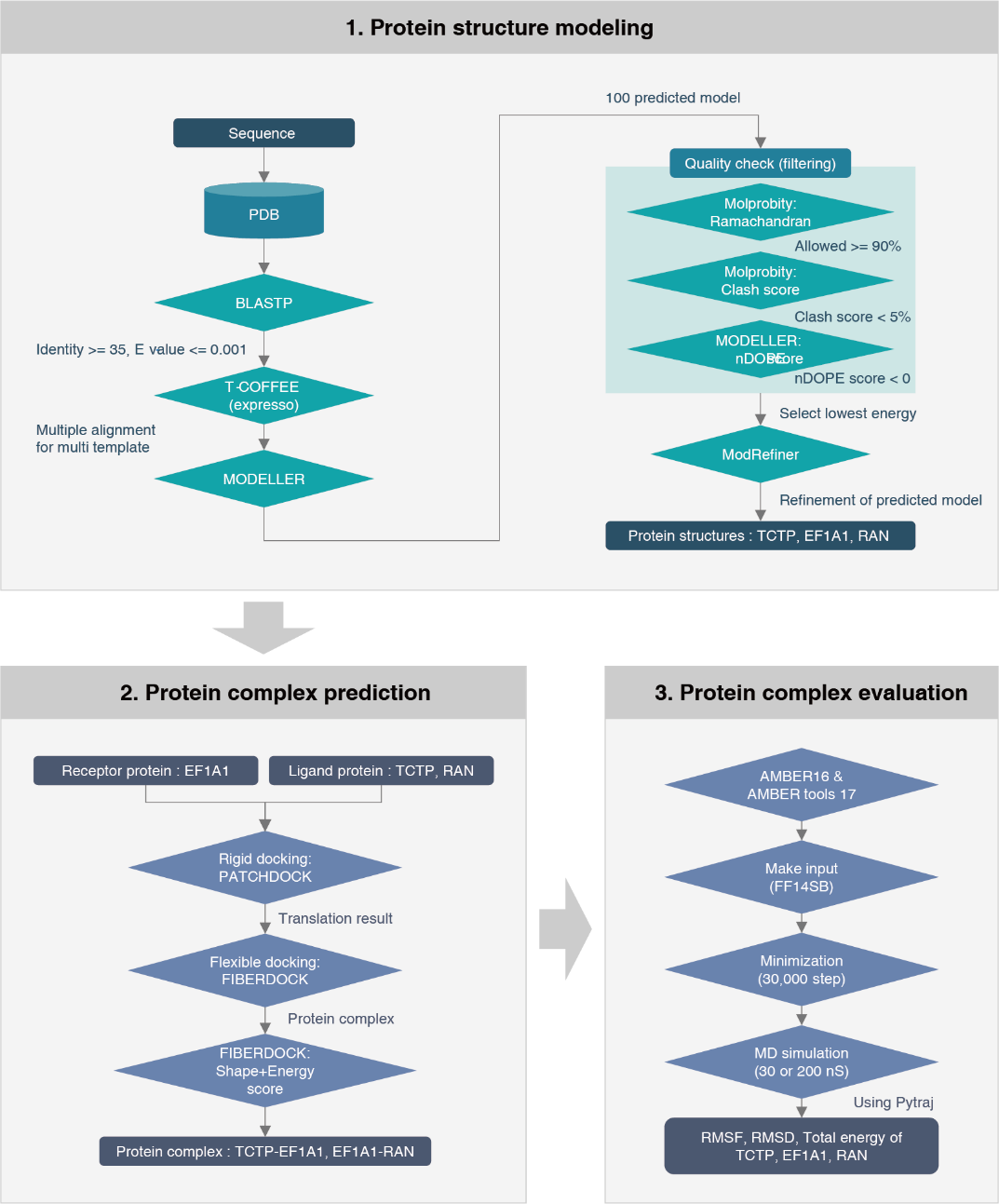
**

**Figure S1. Pipeline for homology modeling and molecular docking of TCTPs and their binding protein.** The pipeline divided into three parts and programs for each part were described in the rectangle with conditions.
