# Supplementary material for: Comprehensive analysis of Translationally Controlled Tumor Protein (TCTP) provides insights for lineage-specific evolution and functional divergence
Source: PLoS One. 2020 May 6;15(5):e0232029. doi: 10.1371/journal.pone.0232029 (PMC7202613; doi:10.1371/journal.pone.0232029)
Supplement: S2 Fig — The distance of each amino acids were measured using human TCTP as a control and average distance and standard deviation of the RMSD were calculated within each organismal divisions. (DOCX) [file pone.0232029.s005.docx]

**
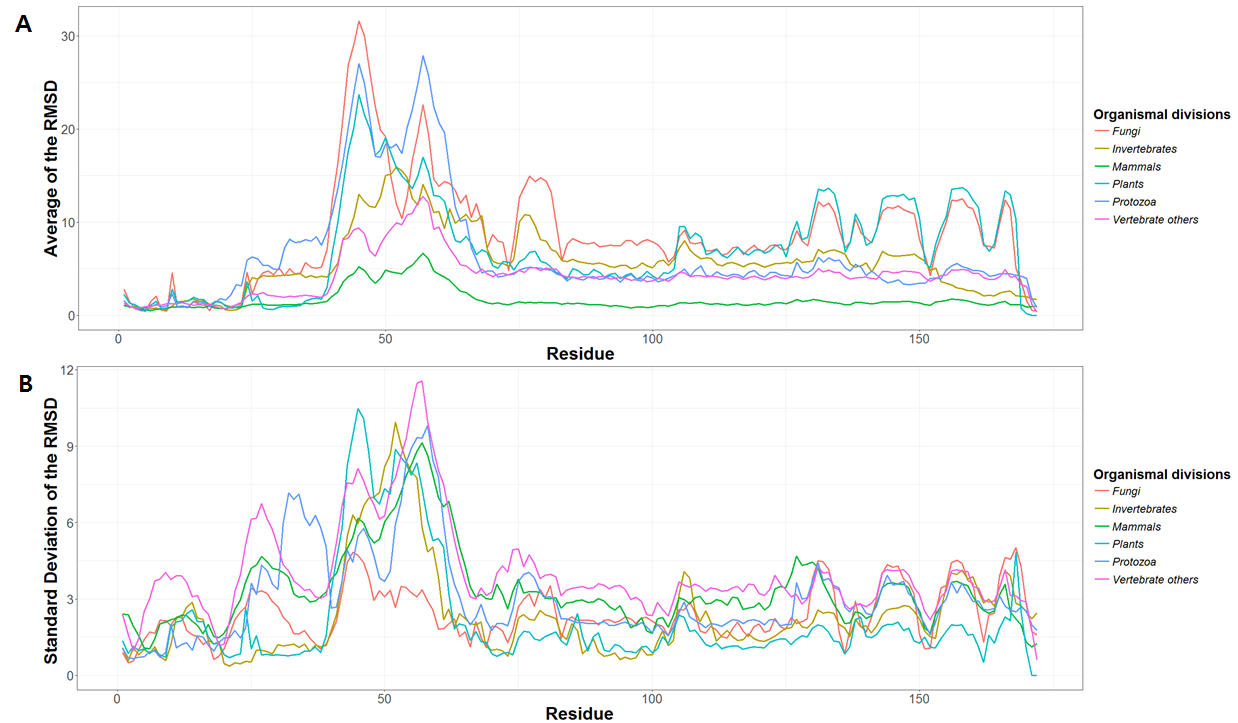
**

**Figure 2. RMSD Distribution of amino acids in individual TCTPs from Human TCTP.** The distance of each amino acids were measured using human TCTP as a control and average distance and standard deviation of the RMSD were calculated within each organismal divisions
