# Supplementary material for: Comprehensive analysis of Translationally Controlled Tumor Protein (TCTP) provides insights for lineage-specific evolution and functional divergence
Source: PLoS One. 2020 May 6;15(5):e0232029. doi: 10.1371/journal.pone.0232029 (PMC7202613; doi:10.1371/journal.pone.0232029)
Supplement: S3 Fig — This figures viewing average secondary structure. To secondary structure predict, we using two method. First, we predicted sequence based secondary structure using psi-pred 3.4. Second, we predicted structure based secondary structure using DSSP. (DOCX) [file pone.0232029.s006.docx]

**
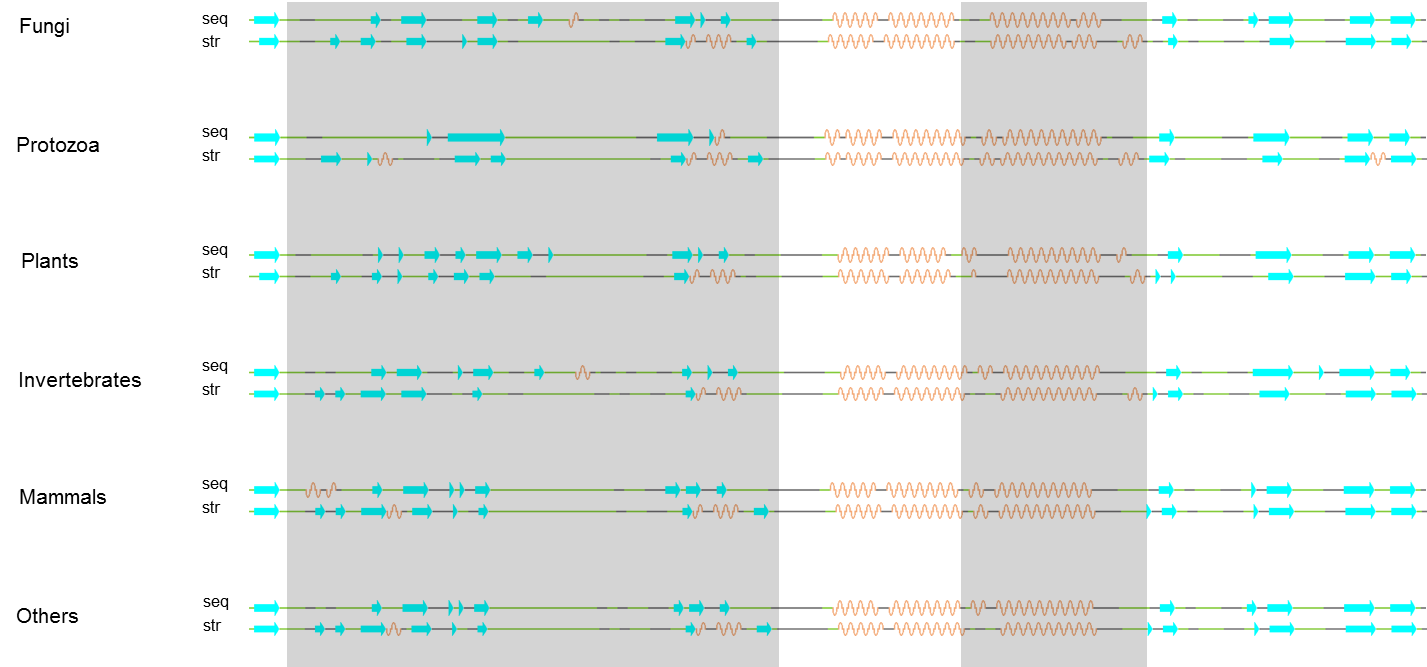
**

**Figure S3. Secondary structure prediction result using two methods.** This figures viewing average secondary structure. To secondary structure predict, we using two method. First, we predicted sequence based secondary structure using psi-pred 3.4. Second, we predicted structure based secondary structure using DSSP.
