# Supplementary material for: Comprehensive analysis of Translationally Controlled Tumor Protein (TCTP) provides insights for lineage-specific evolution and functional divergence
Source: PLoS One. 2020 May 6;15(5):e0232029. doi: 10.1371/journal.pone.0232029 (PMC7202613; doi:10.1371/journal.pone.0232029)
Supplement: S4 Fig — Polarity and secondary structure merging result. (DOCX) [file pone.0232029.s007.docx]

**
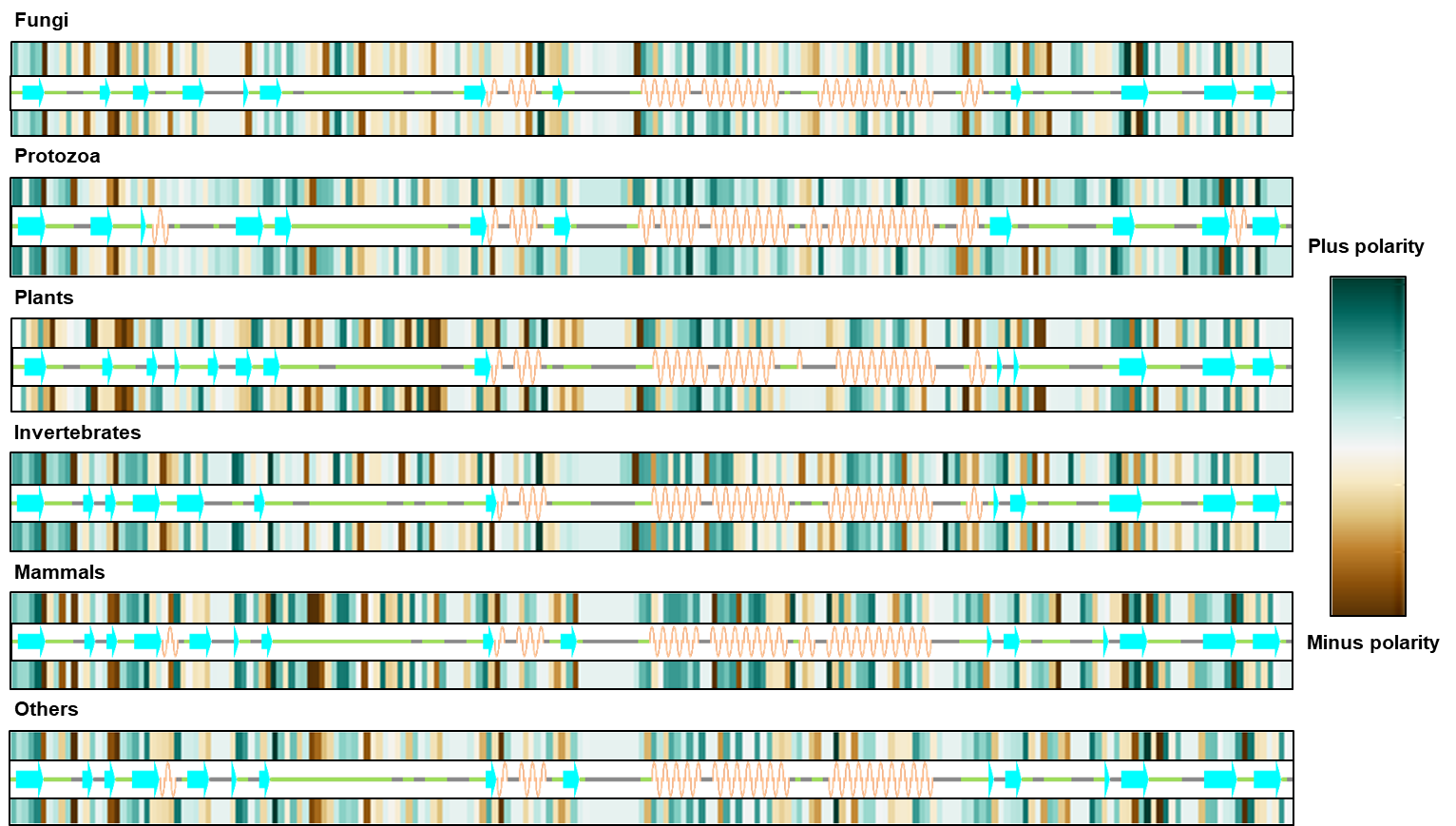
**

**Figure S4. Combined result of multi analysis data.** Polarity and secondary structure merging result.
