# Supplementary material for: Comprehensive analysis of Translationally Controlled Tumor Protein (TCTP) provides insights for lineage-specific evolution and functional divergence
Source: PLoS One. 2020 May 6;15(5):e0232029. doi: 10.1371/journal.pone.0232029 (PMC7202613; doi:10.1371/journal.pone.0232029)
Supplement: S5 Fig — Conservation was assessed in terms of the average conservation rate over all positions in TCTP genes. The conservation score is the proportion of conserved amino acids in a window of size 10. (DOCX) [file pone.0232029.s008.docx]

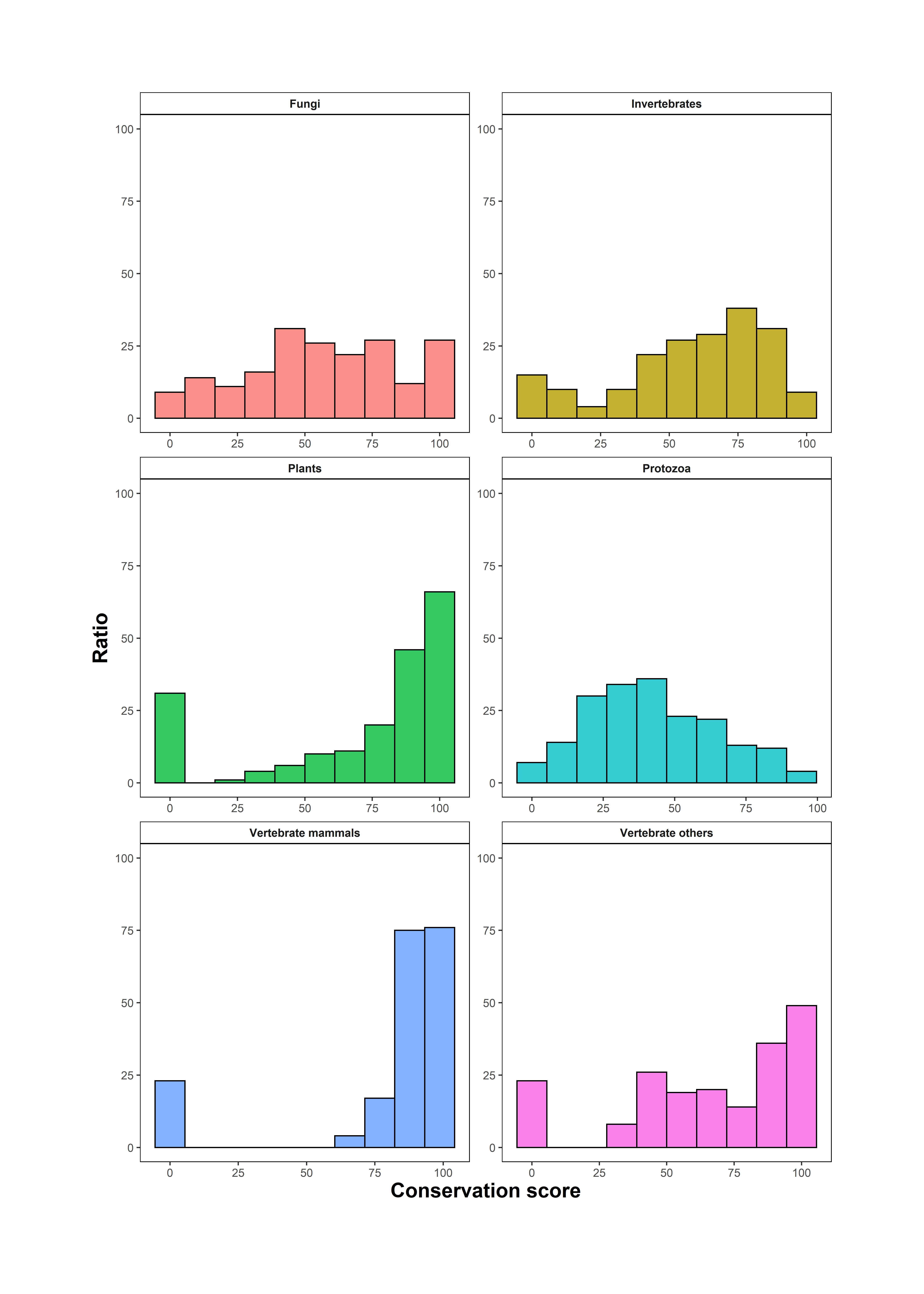


**Figure S5**. **Conserved scores of TCTP genes**. Conservation was assessed in terms of the average conservation rate over all positions in TCTP genes. The conservation score is the proportion of conserved amino acids in a window of size 10.
