# Supplementary material for: Comprehensive analysis of Translationally Controlled Tumor Protein (TCTP) provides insights for lineage-specific evolution and functional divergence
Source: PLoS One. 2020 May 6;15(5):e0232029. doi: 10.1371/journal.pone.0232029 (PMC7202613; doi:10.1371/journal.pone.0232029)
Supplement: S6 Fig — MEPS (left) and hydrophobicity molecular surface (right) of a single turn α-helix and its neighboring region in TCTPs from two representative species were visualized using Chimera. (DOCX) [file pone.0232029.s009.docx]

**
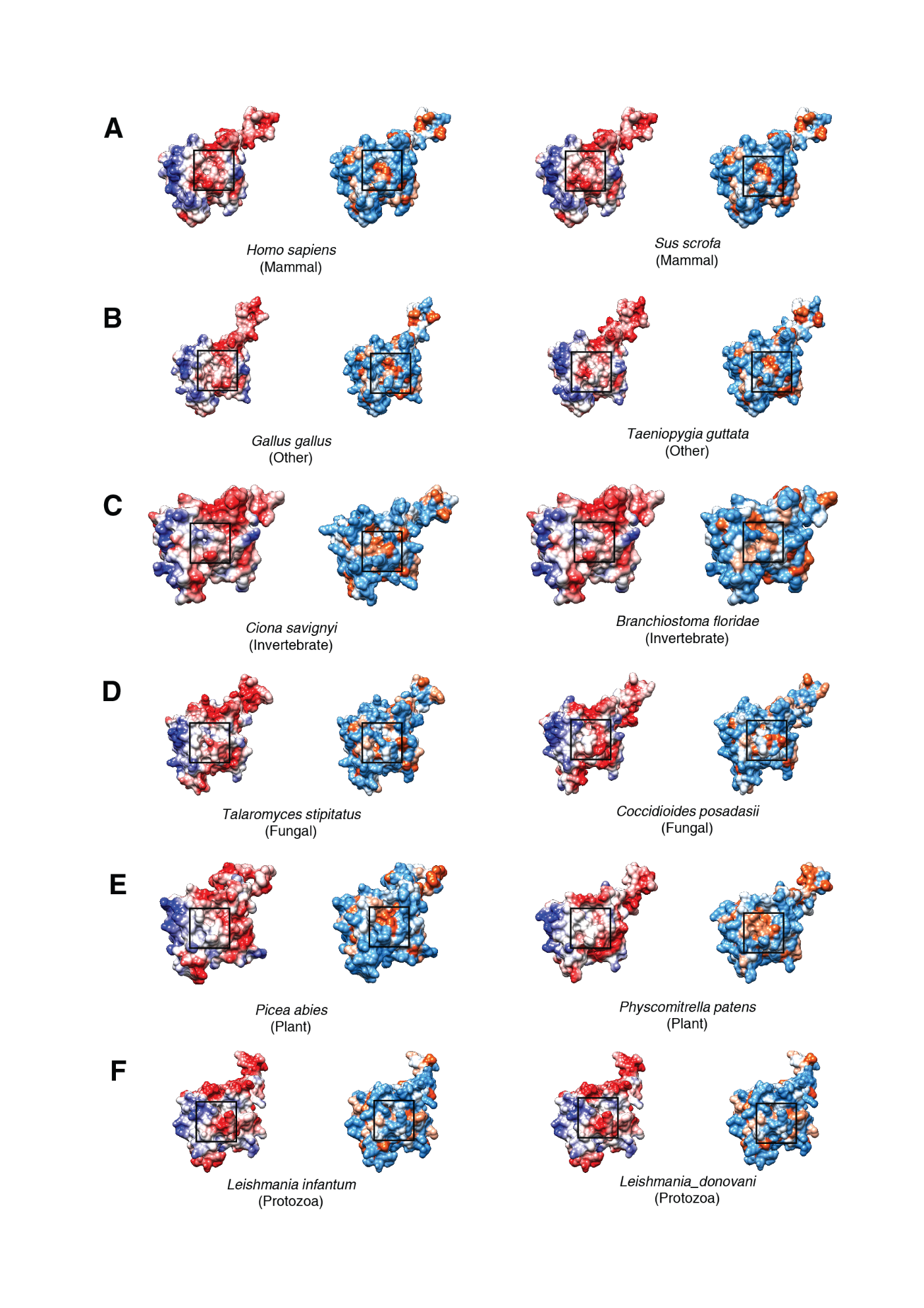
**

**Figure S6. Molecular electrostatic potential surface (MEPS) and hydrophobicity of a single turn α-helix and its neighboring region.** MEPS (left) and hydrophobicity molecular surface (right) of a single turn α-helix and its neighboring region in TCTPs from two representative species were visualized using Chimera
