# Supplementary material for: Comprehensive analysis of Translationally Controlled Tumor Protein (TCTP) provides insights for lineage-specific evolution and functional divergence
Source: PLoS One. 2020 May 6;15(5):e0232029. doi: 10.1371/journal.pone.0232029 (PMC7202613; doi:10.1371/journal.pone.0232029)
Supplement: S7 Fig — Time evolutions of the backbone RMSD of TCTP (A), EF1A1 (B) and RAN(C) were shown. Colored-bars represent corresponding species. (DOCX) [file pone.0232029.s010.docx]

**
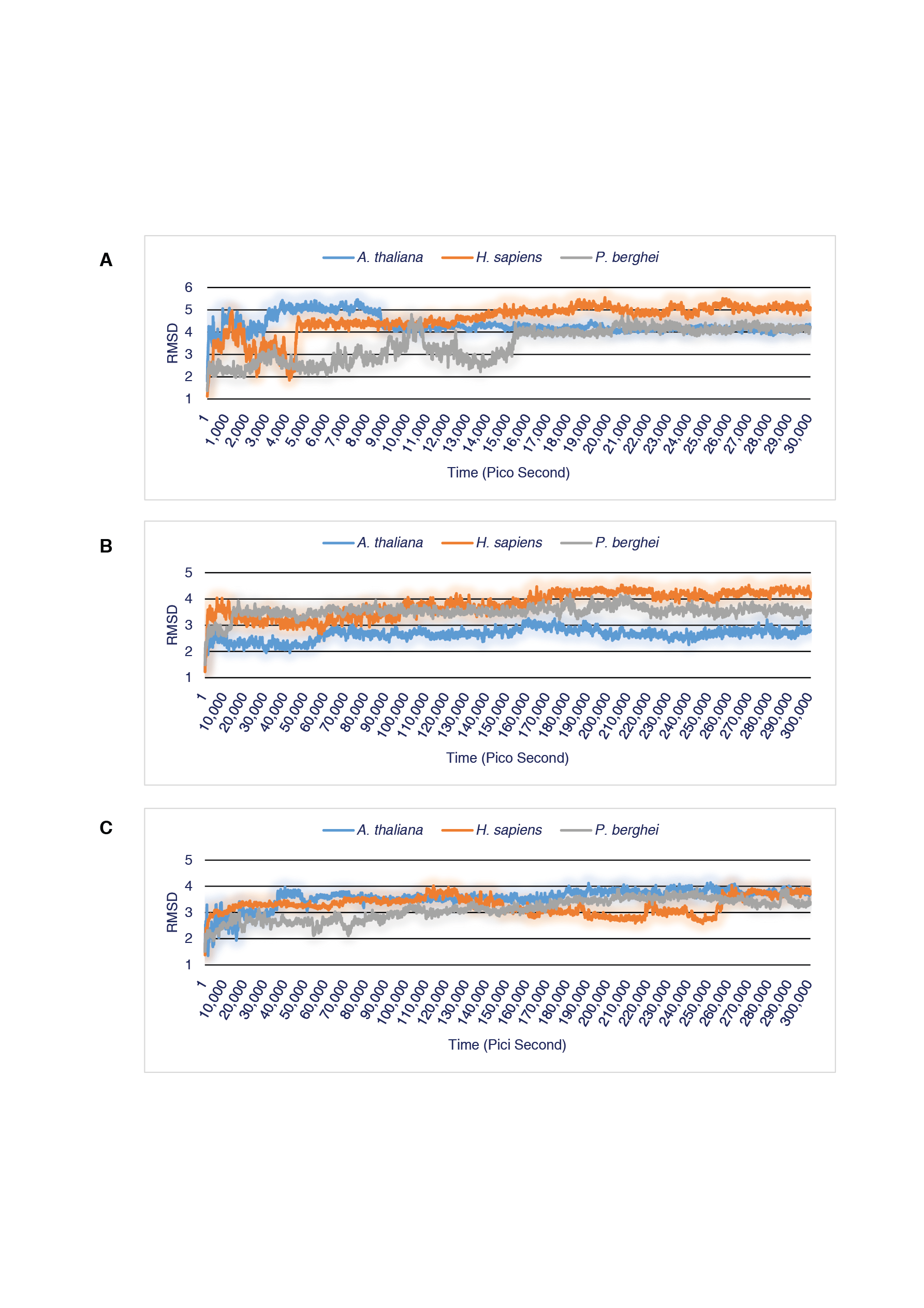
Figure S7. Time variation of the RMSD during the MD simulation period of 30,000 pico second (30 nS) for protein structure (TCTP, EF1A1, RAN) evaluation.** Time evolutions of the backbone RMSD of TCTP (A), EF1A1 (B) and RAN(C) were shown. Colored-bars represent corresponding species.
