# Supplementary material for: Comprehensive analysis of Translationally Controlled Tumor Protein (TCTP) provides insights for lineage-specific evolution and functional divergence
Source: PLoS One. 2020 May 6;15(5):e0232029. doi: 10.1371/journal.pone.0232029 (PMC7202613; doi:10.1371/journal.pone.0232029)
Supplement: S9 Fig — EF1A1-binding region show a flexible property among A. thaliana (A), H. sapiens (B) and P. berghei (C). Purple-colored boxes indicate EF1A1 binding site. (DOCX) [file pone.0232029.s012.docx]

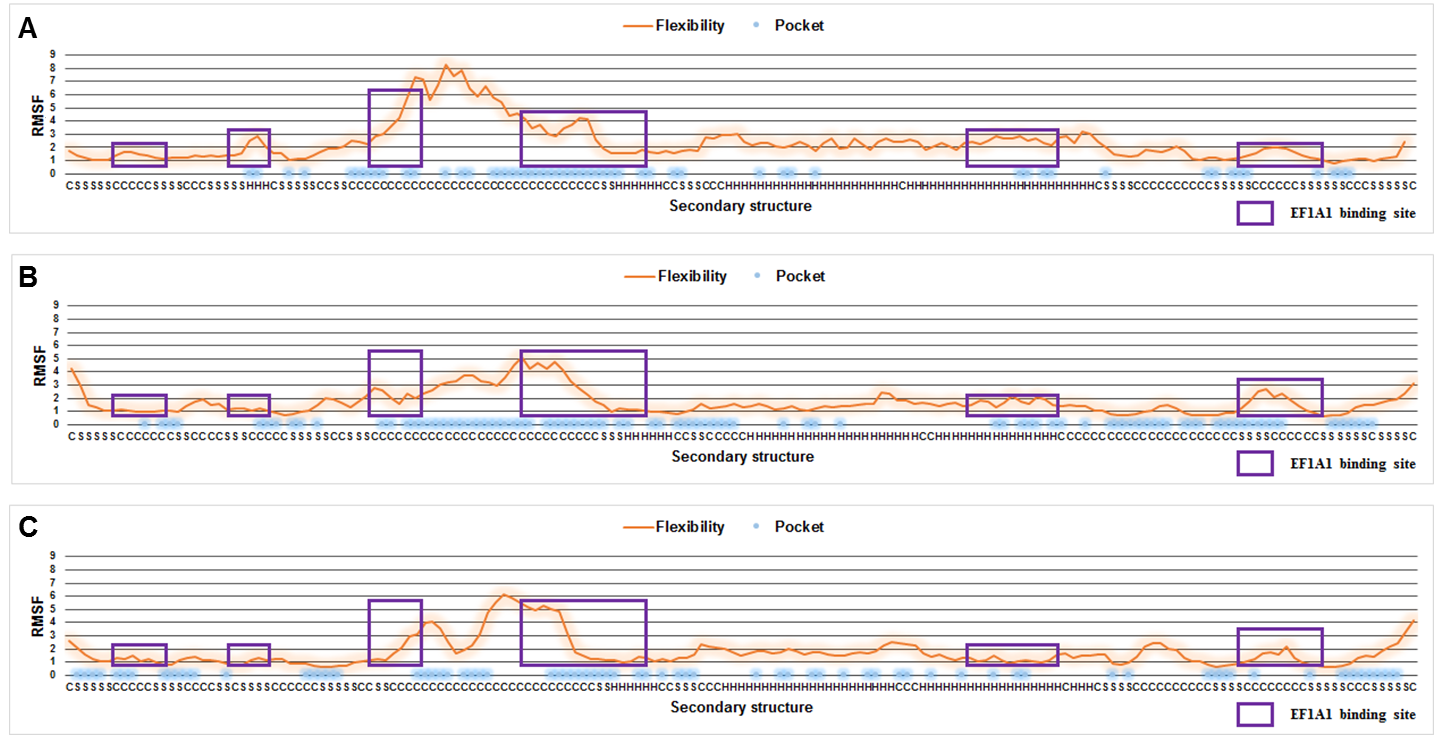


**Figure S9.** **Distribution of RMSF with pocket site for TCTP**. EF1A1-binding region show a flexible property among *A. thaliana* (A), *H. sapiens* (B) and *P. berghei* (C). Purple-colored boxes indicate EF1A1 binding site.
