# Supplementary material for: Comprehensive analysis of Translationally Controlled Tumor Protein (TCTP) provides insights for lineage-specific evolution and functional divergence
Source: PLoS One. 2020 May 6;15(5):e0232029. doi: 10.1371/journal.pone.0232029 (PMC7202613; doi:10.1371/journal.pone.0232029)
Supplement: S10 Fig — The distance matrix of three representative species of TCTP (A), EF1A1 (B), and RAN (C) were shown and red circles represent difference of interacting residues. (DOCX) [file pone.0232029.s013.docx]

**
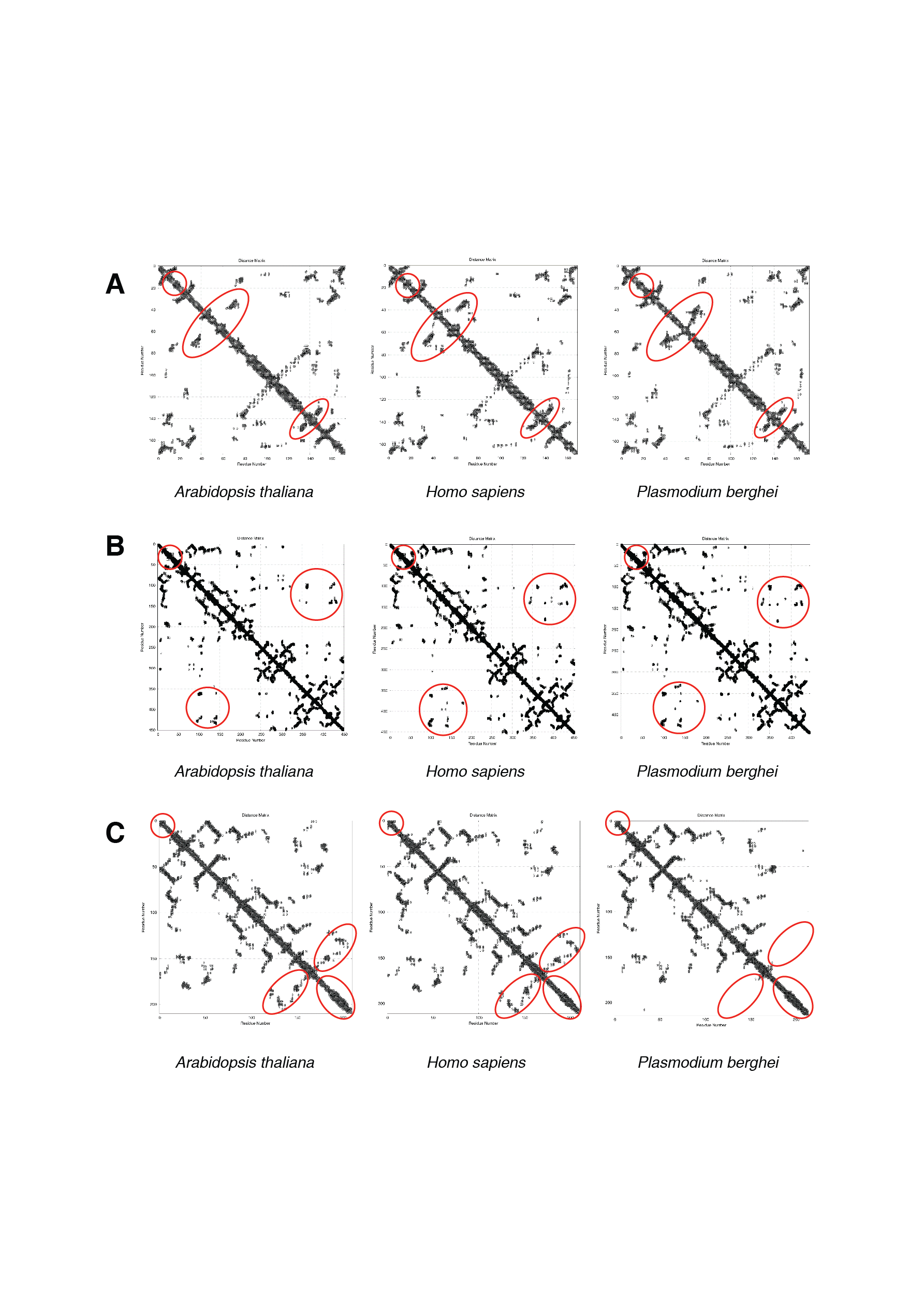
**

**Figure S10. Distance matrix of intra-interaction among residues in a single molecule.** The distance matrix of three representative species of TCTP (A), EF1A1 (B), and RAN (C) were shown and red circles represent difference of interacting residues
