# Supplementary material for: Comprehensive analysis of Translationally Controlled Tumor Protein (TCTP) provides insights for lineage-specific evolution and functional divergence
Source: PLoS One. 2020 May 6;15(5):e0232029. doi: 10.1371/journal.pone.0232029 (PMC7202613; doi:10.1371/journal.pone.0232029)
Supplement: S11 Fig — Time evolutions of (A) total energy of TCTP-EF1A1 and EF1A1-RAN, (B) the backbone RMSD of TCTP-EF1A1 and EF1A1-RAN. Each graph (A, B) include A. thaliana, H. sapiens and P. berghei. (DOCX) [file pone.0232029.s014.docx]

**
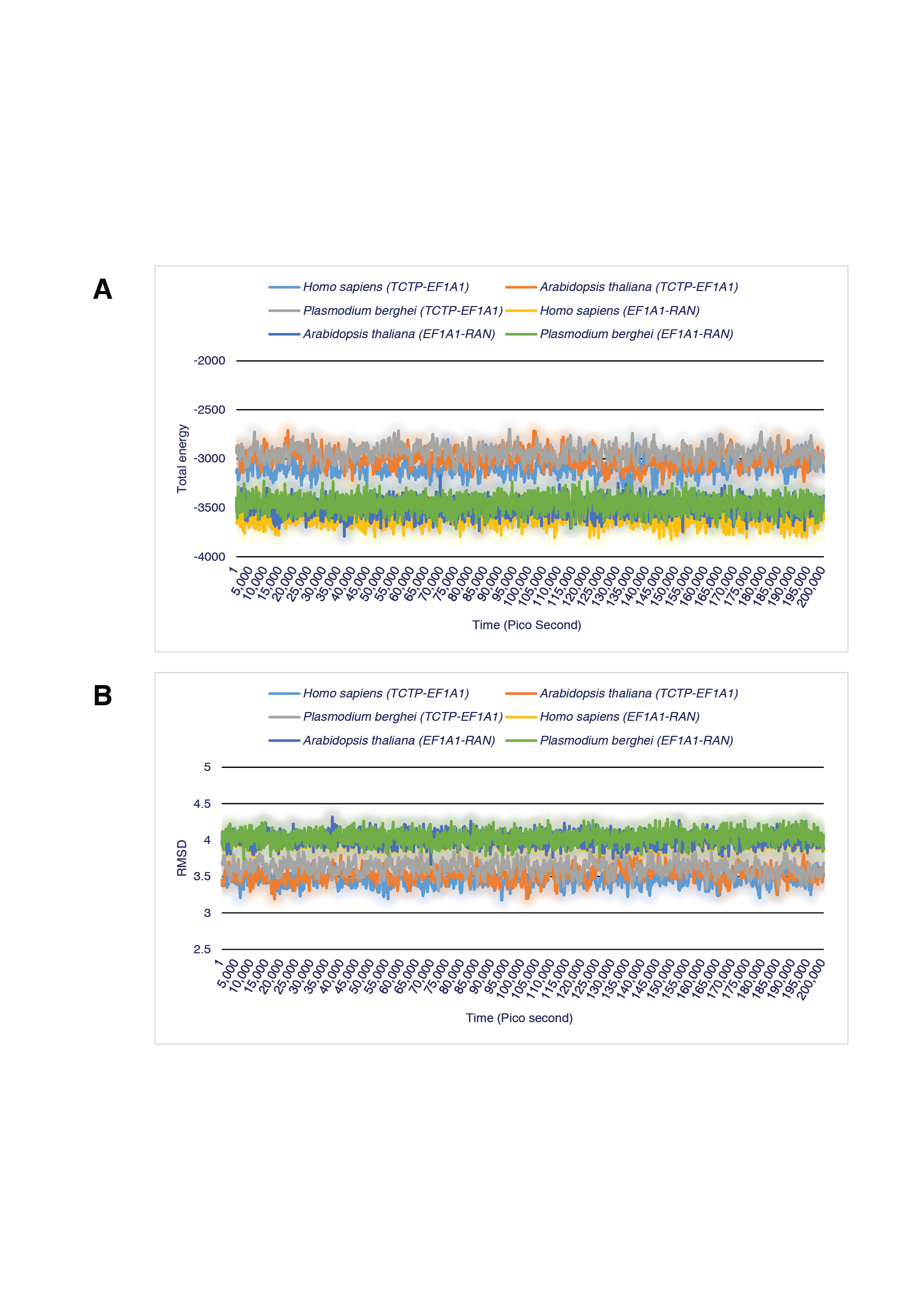
Figure S11. Estimated equilibration state and stability of MD simulation period of 200,000 pico second (200 nS) for complex structure (TCTP-EF1A1, EF1A1-RAN) evaluation.** Time evolutions of (A) total energy of TCTP-EF1A1 and EF1A1-RAN, (B) the backbone RMSD of TCTP-EF1A1 and EF1A1-RAN. Each graph (A, B) include *A. thaliana, H. sapiens* and *P. berghei.*
