# Supplementary material for: Comprehensive analysis of Translationally Controlled Tumor Protein (TCTP) provides insights for lineage-specific evolution and functional divergence
Source: PLoS One. 2020 May 6;15(5):e0232029. doi: 10.1371/journal.pone.0232029 (PMC7202613; doi:10.1371/journal.pone.0232029)
Supplement: S1 Table — (DOCX) [file pone.0232029.s015.docx]

**Table S1.** Structure quality check of predicted protein structure about treat refinement by organismal divisions

|  |  | **Before refinement** | | | **After refinement** | | |
| --- | --- | --- | --- | --- | --- | --- | --- |
| **Gene name** | **Organismal divisions** | ***nDOPE** | ****Clash** | *****Ramachandran** | ***nDOPE** | ****Clash** | *****Ramachandran** |
| **TCTP** | **Plants** | -0.63 | 4.53 | 96.01 | -1.37 | 1.8 | 97.58 |
|  | **Mammals** | -0.19 | 4.32 | 93.27 | -1.16 | 1.45 | 96.05 |
|  | **Fungi** | -0.32 | 3.83 | 93.63 | -1.09 | 1.46 | 96.06 |
|  | **Invertebrates** | -0.43 | 3.83 | 93.73 | -1.18 | 1.52 | 95.91 |
|  | **others** | -0.2 | 4.44 | 93.02 | -1.23 | 1.43 | 95.63 |
|  | **Protozoa** | -0.68 | 3.99 | 96.06 | -1.39 | 1.39 | 97.88 |
| **EF1A1** | **Plants** | -0.71 | 2.85 | 94.47 | -1.26 | 1.22 | 97.91 |
|  | **Mammals** | -0.64 | 2.69 | 95.73 | -1.15 | 1.37 | 98.49 |
|  | **Fungi** | -0.55 | 5.19 | 92.88 | -1.2 | 1.25 | 97.45 |
|  | **Invertebrates** | -0.99 | 3.79 | 94.53 | -1.55 | 1.18 | 97.81 |
|  | **others** | -0.8 | 1.78 | 96 | -1.33 | 1.14 | 98.47 |
|  | **Protozoa** | -0.8 | 3.98 | 94.27 | -1.48 | 1.36 | 97.49 |
| **RAN** | **Plants** | -0.74 | 1.53 | 95.02 | -1.34 | 0.71 | 97.36 |
|  | **Mammals** | -0.56 | 2.68 | 94.29 | -1.11 | 1.12 | 97.22 |
|  | **Fungi** | -1.1 | 1.18 | 95.69 | -1.45 | 0.67 | 97.58 |
|  | **Invertebrates** | -0.66 | 2.88 | 94.34 | -1.15 | 1.21 | 96.89 |
|  | **others** | -1.08 | 1.13 | 95.68 | -1.45 | 0.62 | 97.58 |
|  | **Protozoa** | -1.11 | 1.22 | 95.9 | -1.52 | 0.68 | 97.64 |

* Energy stability score: Normalized dope score in modeller package

**Clash score: clash-score describes the clashes present in a protein-structure in molprobity package

***Ramachandran score: Ramachandran score relative to current state-of-the-art structures
