# Supplementary material for: Comprehensive analysis of Translationally Controlled Tumor Protein (TCTP) provides insights for lineage-specific evolution and functional divergence
Source: PLoS One. 2020 May 6;15(5):e0232029. doi: 10.1371/journal.pone.0232029 (PMC7202613; doi:10.1371/journal.pone.0232029)
Supplement: S2 Table — (DOCX) [file pone.0232029.s016.docx]

**Table S2.** Sub-chain number in the selected templates by each organismal division

| **Organismal divisions** | **1h6q^1^** | **1h7y^1^** | **1txj^2^** | **1yz1^2^** | **2hr9^2^** | **2kwb^2^** | **2loy^3^** | **3ebm^3^** | **3p3k^4^** | **4z9v^5^** |
| --- | --- | --- | --- | --- | --- | --- | --- | --- | --- | --- |
| **Mammals** | 11% | 11% | 6% | 11% | 11% | 9% | 9% | 11% | 10% | 11% |
| **Vertebrate others** | 12% | 12% | 5% | 12% | 12% | 10% | 10% | 12% | 8% | 10% |
| **Invertebrates** | 13% | 13% | 6% | 12% | 15% | 13% | 13% | 12% | 3% | 0% |
| **Plants** | 22% | 22% | 1% | 2% | 23% | 14% | 14% | 1% | 1% | 0% |
| **Protozoa** | 20% | 20% | 13% | 8% | 12% | 2% | 2% | 13% | 12% | 0% |
| **Fungi** | 17% | 17% | 7% | 16% | 17% | 4% | 4% | 15% | 3% | 0% |

^1^ *Schizosaccharomyces pombe*

^2^ *Homo sapiens*

^3^ *Caenorhabditis elegans*

^4^ *Plasmodium knowlesi*

^5^ *Plasmodium falciparum*
