# Supplementary material for: Comprehensive analysis of Translationally Controlled Tumor Protein (TCTP) provides insights for lineage-specific evolution and functional divergence
Source: PLoS One. 2020 May 6;15(5):e0232029. doi: 10.1371/journal.pone.0232029 (PMC7202613; doi:10.1371/journal.pone.0232029)
Supplement: S3 Table — (DOCX) [file pone.0232029.s017.docx]

**Table S3.** Structure quality check between modeling structure and PDB

| PDB ID | Organismal division | Species | General predicted model | | | Refined model | | | PDB structure | | |
| --- | --- | --- | --- | --- | --- | --- | --- | --- | --- | --- | --- |
|  |  |  | nDOPE | Clash | Ramachandran | nDOPE | Clash | Ramachandran | nDOPE | Clash | Ramachandran |
| 2hr9 | Mammal | *Homo sapiens* | -0.298 | 4.36 | 92 | -1.302 | 0.85 | 95 | -0.937 | 0.96 | 91 |
| 2loy | Invertebrate | *Caenorhabditis elegans* | -0.723 | 3.68 | 96 | -1.519 | 1.5 | 98 | -0.71 | 0.47 | 87 |
| 1h6q | Fungal | *Schizosaccharomyces pombe* | -0.934 | 3.28 | 97 | -1.536 | 1.26 | 99 | -0.344 | 2.14 | 85 |
| 1h7y | Fungal | *Schizosaccharomyces pombe* | -0.934 | 3.28 | 97 | -1.536 | 1.26 | 99 | -0.344 | 2.08 | 85 |
| 1txj | Protozoan | *Plasmodium knowlesi* | -1.217 | 2.38 | 98 | -1.814 | 0.73 | 99 | -2.016 | 0.18 | 99 |
| 2kwb | Invertebrate | *Caenorhabditis elegans* | -0.961 | 1.91 | 96 | -1.342 | 0.97 | 97 | -0.596 | 0.8 | 89 |
| 1yz1 | Mammal | *Homo sapiens* | -0.298 | 4.36 | 92 | -1.302 | 0.85 | 95 | -2.049 | 0.43 | 98 |
| 3ebm | Mammal | *Homo sapiens* | -0.298 | 4.36 | 92 | -1.302 | 0.85 | 95 | -1.892 | 0.21 | 98 |
| 3p3k | Protozoan | *Plasmodium falciparum* | -1.066 | 3.06 | 98 | -1.613 | 0.98 | 99 | -1.733 | 0.05 | 99 |

* Yellow box means best result in each score ranking
