# Supplementary material for: Comprehensive analysis of Translationally Controlled Tumor Protein (TCTP) provides insights for lineage-specific evolution and functional divergence
Source: PLoS One. 2020 May 6;15(5):e0232029. doi: 10.1371/journal.pone.0232029 (PMC7202613; doi:10.1371/journal.pone.0232029)
Supplement: S4 Table — (DOCX) [file pone.0232029.s018.docx]

**Table S4.** Structural clustering of TCTP proteins using MaxCluster program

| **Cluster** | **Centroid*** | **Numbers**** | **Spread***** | **Structure ID** |
| --- | --- | --- | --- | --- |
| 3 | 3 | 39 | 0.274 | Fungi Group 1 |
| 8 | 4 | 12 | 0.321 | Fungi Group 3 |
| 6 | 7 | 24 | 0.371 | Fungi Group 2 |
| 13 | 92 | 6 | 0.292 | Fungi Group 4 |
| 14 | 129 | 5 | 0.153 | Invertebrates Group 2 |
| 5 | 130 | 25 | 0.227 | Invertebrates Group 1 |
| 7 | 187 | 22 | 0.301 | Plants Group 2 |
| 2 | 228 | 92 | 0.32 | Plants Group 1 |
| 9 | 335 | 11 | 0.201 | Protozoa Group 1 |
| 10 | 338 | 11 | 0.15 | Protozoa Group 2 |
| 4 | 409 | 26 | 0.148 | Mammals Group 2 |
| 15 | 454 | 5 | 0.318 | Mammals Group 4 |
| 12 | 542 | 10 | 0.355 | Mammals Group 3 |
| 1 | 576 | 147 | 0.237 | Mammals Group 1,  Vertebrate others Group 1 |
| 11 | 591 | 11 | 0.166 | Vertebrate others Group 2 |

* Centroid: Assigned number of representative protein in each cluster

**Numbers: Numbers of proteins in each cluster

***Spread: Average of distance based on protein structure similarity
