# Supplementary material for: Comprehensive analysis of Translationally Controlled Tumor Protein (TCTP) provides insights for lineage-specific evolution and functional divergence
Source: PLoS One. 2020 May 6;15(5):e0232029. doi: 10.1371/journal.pone.0232029 (PMC7202613; doi:10.1371/journal.pone.0232029)
Supplement: S5 Table — (DOCX) [file pone.0232029.s019.docx]

**Table S5. Comparison of structural similarity (TMalign score) by organismal deviations.**

| **Organismal divisions (**number of proteins**)** | **Fungi** | **Invertebrates** | **Plants** | **Protozoa** | **Mammals** | **Vertebrate others** |
| --- | --- | --- | --- | --- | --- | --- |
| **Fungi** | 0.87 |  |  |  |  |  |
| **Invertebrates** | 0.85 | 0.91 |  |  |  |  |
| **Plants** | 0.84 | 0.82 | 1.00 |  |  |  |
| **Protozoa** | 0.81 | 0.81 | 0.79 | 0.84 |  |  |
| **Mammals** | 0.80 | 0.80 | 0.78 | 0.77 | 0.92 |  |
| **Vertebrate others** | 0.76 | 0.77 | 0.73 | 0.72 | 0.81 | 0.88 |
