# Supplementary material for: Comprehensive analysis of Translationally Controlled Tumor Protein (TCTP) provides insights for lineage-specific evolution and functional divergence
Source: PLoS One. 2020 May 6;15(5):e0232029. doi: 10.1371/journal.pone.0232029 (PMC7202613; doi:10.1371/journal.pone.0232029)
Supplement: S6 Table — (DOCX) [file pone.0232029.s020.docx]

**Table S6.** Structural similarity score of TCTP, EF1A1 and RAN

| **Gene name** | **Fungi** | **Invertebrates** | **Plants** | **Protozoa** | **Mammals** | **Vertebrate  others** |
| --- | --- | --- | --- | --- | --- | --- |
| **TCTP** | 81.61 (118/118, 124) | 83.46 (48/48, 57) | 80.9 (53/53, 148) | 81.28 (46/46, 56) | 92.3 (75/75, 191) | 88.88 (39/39, 48) |
| **eEF1A1** | 93.4 (50/118, 258) | 94.83 (29/48, 119) | 92.37 (35/53, 319) | 90.84 (40/46, 403) | 96.47 (66/75, 477) | 97.3 (35/39, 172) |
| **RAN** | 88.51 (19/118, 33) | 87.85 (26/48, 53) | 85.16 (34/53, 115) | 85.31 (27/46, 109) | 90.77 (64/75, 178) | 90.98 (35/39, 57) |

* Average of TM score (number of species containing genes of interests /number of species containing TCTP gene, number of proteins)
