# Supplementary material for: Comprehensive analysis of Translationally Controlled Tumor Protein (TCTP) provides insights for lineage-specific evolution and functional divergence
Source: PLoS One. 2020 May 6;15(5):e0232029. doi: 10.1371/journal.pone.0232029 (PMC7202613; doi:10.1371/journal.pone.0232029)
Supplement: S7 Table — (DOCX) [file pone.0232029.s021.docx]

**Table S7**. Structure quality check of representative EF1A1 protein structure by Organismal Divisions

| **Organismal divisions** | **Species** | **Protein ID** | **Energy stability score** | **Clash score**** | | | **Ramachandran score***** | | |
| --- | --- | --- | --- | --- | --- | --- | --- | --- | --- |
|  |  |  | **nDOPE score*** | **Clash atom number** | **Total atom number** | **Clash percentage** | **Favored** | **Allowed** | **Outlier** |
| **Fungi** | *Coprinopsis cinerea* | XP_001828758.1 | -1.344 | 20.27 | 3453 | 0.59 | 436 | 10 | 4 |
| **Invertebrates** | *Drosophila melanogaster* | NP_477375.1 | -1.22 | 42.97 | 3328 | 1.29 | 414 | 9 | 2 |
| **Plants** | *Arabidopsis thaliana* | NP_563801.1 | -1.205 | 31.88 | 3419 | 0.93 | 425 | 13 | 1 |
| **Protozoa** | *Plasmodium berghei* | XP_677917.1 | -1.289 | 25.37 | 3390 | 0.75 | 427 | 11 | 1 |
| **Mammals** | *Homo sapiens* | NP_001393.1 | -1.377 | 23.02 | 3475 | 0.66 | 435 | 11 | 5 |
| **Vertebrate others** | *Alligator mississippiensis* | XP_006269814.1 | -1.194 | 25.17 | 3457 | 0.73 | 436 | 12 | 3 |

* Energy stability score: Normalized dope score in modeller package

**Clash score: clash-score describes the clashes present in a protein-structure in molprobity package

***Ramachandran score: Ramachandran score relative to current state-of-the-art structures
