# Supplementary material for: Comprehensive analysis of Translationally Controlled Tumor Protein (TCTP) provides insights for lineage-specific evolution and functional divergence
Source: PLoS One. 2020 May 6;15(5):e0232029. doi: 10.1371/journal.pone.0232029 (PMC7202613; doi:10.1371/journal.pone.0232029)
Supplement: S8 Table — (DOCX) [file pone.0232029.s022.docx]

**Table S8**. Structure quality check of representative RAN protein structure by Organismal Divisions

| **Organismal divisions** | **Species** | **Protein ID** | **Energy stability score** | **Crash score**** | | | **Ramachandran score***** | | |
| --- | --- | --- | --- | --- | --- | --- | --- | --- | --- |
|  |  |  | **nDOPE score*** | **Clash atom number** | **Total atom number** | **Clash percentage** | **Favored** | **Allowed** | **Outlier** |
| **Fungi** | *Coprinopsis cinerea* | XP_001833111.1 | -0.998 | 16.5 | 1697 | 0.97 | 203 | 6 | 2 |
| **Invertebrates** | *Drosophila melanogaster* | NP_651969.1 | -0.944 | 52.17 | 1725 | 3.02 | 199 | 10 | 4 |
| **Plants** | *Arabidopsis thaliana* | NP_197501.1 | -0.741 | 20.98 | 1668 | 1.26 | 195 | 5 | 6 |
| **Protozoa** | *Plasmodium berghei* | XP_678806.1 | -1.109 | 57.7 | 1681 | 3.43 | 194 | 8 | 1 |
| **Mammals** | *Homo sapiens* | NP_006316.1 | -1.233 | 18.43 | 1682 | 1.1 | 203 | 4 | 1 |
| **Vertebrate  others** | *Alligator mississippiensis* | XP_006268016.1 | -0.834 | 15.73 | 1716 | 0.92 | 208 | 4 | 1 |

* Energy stability score: Normalized dope score in modeller package

**Clash score: clash-score describes the clashes present in a protein-structure in molprobity package

***Ramachandran score: Ramachandran score relative to current state-of-the-art structures
