# Supplementary material for: Comprehensive analysis of Translationally Controlled Tumor Protein (TCTP) provides insights for lineage-specific evolution and functional divergence
Source: PLoS One. 2020 May 6;15(5):e0232029. doi: 10.1371/journal.pone.0232029 (PMC7202613; doi:10.1371/journal.pone.0232029)
Supplement: S9 Table — (DOCX) [file pone.0232029.s023.docx]

**Table S9.** Statistics of secondary structure among 3, 4 ß-sheet

| **Organismal divisions** | **α-helix** | | | | | | **Turn (Coil)** | | | | | **Total numbers of proteins**  **(A+B)** |
| --- | --- | --- | --- | --- | --- | --- | --- | --- | --- | --- | --- | --- |
|  | **Numbers of** | | **Best template** | | | | **Numbers of** | | **Best template** | | |  |
|  | **proteins**  **(A)** | | **α-helix** | | **Turn (Coil)** | | **proteins**  **(B)** | | **α-helix** | | **Turn (Coil)** |  |
| **Fungi** | 9 | 1 | | 8 | | 115 | | 1 | | 114 | | 124 |
| **Invertebrates** | 3 | 1 | | 2 | | 54 | | 1 | | 53 | | 57 |
| **Plants** | 26 | 2 | | 24 | | 121 | | 7 | | 114 | | 147 |
| **Protozoa** | 19 | 8 | | 11 | | 39 | | 10 | | 29 | | 58 |
| **Mammals** | 136 | 5 | | 131 | | 43 | | 3 | | 40 | | 179 |
| **Others** | 39 | 2 | | 37 | | 9 | | 0 | | 9 | | 48 |
